# Quantum Leaps in Human Biocultural Evolution and the Relationship to Cranial Capacity

**DOI:** 10.3390/life13041030

**Published:** 2023-04-17

**Authors:** Gerhard W. Weber

**Affiliations:** 1 Department of Evolutionary Anthropology, University of Vienna, 1030 Vienna, Austria; gerhard.weber@univie.ac.at; Tel.: +43-1-4277-54701; 2 Human Evolution and Archaeological Sciences (HEAS), University of Vienna, 1030 Vienna, Austria

**Keywords:** biocultural evolution, cranial capacity, cognitive performance, stone tools, symbolic behavior, intentional evolution, posthumanism

## Abstract

The evolution of the genus *Homo* can only be understood by considering both of the inheritance systems that interact to shape human nature: biology and culture. While growing intellectual abilities are a key factor of human evolution, they are rarely contrasted with cultural progress. Cranial capacity data of 193 hominin fossils from the last seven million years and artefacts of increasing number and complexity in the archaeological record are used to demonstrate the concordant progression of brain-size increase and cultural development, starting approximately two million years ago. Our biocultural evolution shows a number of quantum leaps along the time axis applying to both domains. At first, humans left the canonical evolutionary pathway, which pertains to all other organisms, by enhancing their fitness using sophisticated tools and fire; secondly, they turned into a symbolic species; and finally, humanity now faces a new challenge: “intentional evolution”. Chronologically, these quantum leaps correspond to cranial capacity data used here as a proxy for cognitive performance. This contribution tries to demonstrate this parallel development and argues for a simple and generalized model of human biocultural evolution. An extrapolation of the model into the future shows that humans, as biological entities, will not necessarily persist.

## 1. Introduction

Human evolution of the last million years was not a mere biological process but, to an increasing extent, also an act of cultural development [1]. Although humans are fundamentally biological creatures, we have been able to gradually attenuate, or even eliminate, restraints on the survival of our species through increasing brain size and cognitive abilities, which enabled the progressive development of a second inheritance system commonly referred to as “culture”. This second system builds upon the foundations of the primary one—genes—and shows many parallel aspects such as innovation, variation, selection of the best adapted elements, and inheritance [2]. Cultural transmission is, however, also profoundly different from genetic transmission, for instance, in its possibility of horizontal or oblique diffusion among nonrelatives from different generations, in its dynamics, which can be quite fast [3], and in its possible non-randomness, if intelligence is exploited to reach intended goals. The interaction of these two evolutionary modes, organic and cultural, already recognized by Darwin [4,5], was intensively explored in the 1970s and 1980s [6,7] and certainly does not apply to humans only. The existing body of literature in biocultural evolution, or gene-culture coevolution, concerned with the study of complex interactions between the two systems in humans [8], other vertebrates, or insects, is impressive, and a summary is beyond the scope of this contribution (a collection of dedicated articles can, e.g., be found in a special PNAS issue [9], Vol 114, no. 30, 2017). While it is increasingly recognized that social learning and cultural transmission are quite widespread in the animal kingdom, the evolution of *Homo* is strongly and increasingly associated with cumulative cultural inheritance [10,11]. Despite the still ongoing debate about whether non-human species such as chimpanzees, crows, or macaques have developed cumulative cultural skills [12,13], we can clearly observe that humans outperform all other creatures of the animal kingdom in this competence.

Humans became extraordinarily effective in transforming their own culture as well as their environment. There is a complex network of reciprocal feedbacks between organisms and their environments [14]. In the early 2000s, organism-driven environmental modifications were considered an additional evolutionary force, known as niche construction, which generates feedback in the form of altered energy and nutrient flow through ecosystems [15]. Based on growing cognitive skills, humanity developed an outstanding ecological plasticity, occupying extreme habitats from high latitudes and high altitudes to open dry steppe and dense rain forests. Some authors argue that our species developed a new ecological niche, that of the “generalist specialist”, even several hundred thousand years ago during the Middle-Late Pleistocene [16].

“Reasoning”, which Schopenhauer defined perhaps too narrowly when he stated that recognizing causality is the mind’s only function, its sole strength [17], has changed the game of our (and other creatures’) evolution and is clearly related to the cognitive capacity of the brain. The increase in cranial capacity in the course of hominin evolution is well documented and was recognized as one of the most distinctive features of humans compared to other evolving creatures (e.g., [18,19,20,21]; see more references in Supplementary Information). The “mind” is a complex phenomenon resulting from the cognitive abilities of a creature and includes observing, learning, recalling, imagining, fantasizing, and all forms of thinking from reflecting, deciding, and planning to anticipating, assessing, and controlling. While we recognize that causality is definitely not restricted to the human mind, causal and probabilistic reasoning in humans includes additional dimensions: consciousness and reflection. It is difficult to demonstrate when these abilities developed on the way from ape-like Miocene hominins to Pleistocene *Homo*, but it is not so difficult to connect technological innovations such as advanced stone tools (e.g., Acheulean hand axes) or the use of fire—which go far beyond anything that animals are able to produce—with growing cranial capacity and intellectual capabilities. In doing so, the mere number and diversity of objects included from the abiotic domain into daily life was progressively increasing. Using extracorporeal aids for survival is again quite common in the animal kingdom—for instance, a spider’s web, a bird’s nest, a beaver’s dam. Nevertheless, the quantity and complexity of extracorporeal devices developed by humans is as impressive as the shifting proportion of current human abilities from those that are physically innate (e.g., walking and running) to those that are culturally acquired (e.g., driving, diving, flying). We extended our phenotype [22] more radically than any other species beyond our inherited physical traits. Claiming that the role of the human mind in evolution has reached an unprecedented level in the animal kingdom does not imply any teleological meaning. For the natural scientist, it is not necessary to imply any goal or purpose behind evolution.

However, compelling questions in the context of *Homo* evolution can be easily identified:

- Since when can we recognize the influence of cognitive abilities on human survival strategies going clearly beyond those of other animals?
- Was this a relatively continuous process, or can we identify leaps?
- Can we diagnose a concordance between brain evolution and cultural developments?

The application of the term “leap” is indeed vulnerable. Recognizing continuous (slow) development, or instead fast transformation (leaps), is dependent on the magnitude of change and the time needed to move from one state to another. For instance, the astounding progression in information technology and genetics of the last 100 years was actually a continuous one, leading from one little achievement to the next one, but in a rather short time. Viewed by an archaeologist in 40,000 years, it would, however, appear as a sudden giant leap because the time resolution and the available data would likely not be sufficient to reproduce the million little steps in this development. Viewed ex post, major changes in history will appear as leaps. The appraisal of “magnitude of transformation per time” remains subjective because there is no objective scale for it, similarly as there is no metric scale for cultural complexity (see below). 

Cognitive performance and cultural achievements can certainly not be related one to one. However, on a larger scale, looking far back in time, their roughly parallel development becomes obvious. In this contribution, cranial capacity data from hominin fossils of the last seven million years, a biological proxy for cognitive abilities, and dating results are re-evaluated. These data are metric; volumes can be measured in milliliters (mL) and age in years (a). However, we do not have good objective measures for overall cultural complexity; there is no metric scale to assess the value of a hand axe from the Early Acheulean compared to an ivory figurine from the Upper Paleolithic. We can just observe the phenomena which appear over time and in some instances recognize the achievement of new qualities—or quantum leaps—in cultural behavior in the archaeological record. The aim here is to contrast biological with cultural developments to generate a simple model of human biocultural evolution, which is currently missing. 

Because of the exponential growth of life—and also successful technologies—quantum leaps, or better, their beginnings, are very difficult to detect, while it is much easier to recognize them when they already happened long ago. In archaeology, we have an additional hurdle: the scarcity of remains. Exponential development starts out almost imperceptibly and then explodes with unexpected fury [23]. How improbable is it to find traces of new advancements *at their beginning* when we look into the deep past? We can assume in most instances that when we luckily find remains—fossils or artefacts—their kind had long been established before they were abundant, and out of the thousands or millions, one specimen was preserved, and we had the fortune to discover it. Still, we may recognize a rapid increase in different but related items pointing in the same direction of development, and we may see the achievement of new levels of complexity. 

This is where the present paper tries to make a contribution. It addresses the few quantum leaps in our cultural evolution and connects them with the apparent biological driving force behind them—the increasing human cognitive capabilities. We are confronted with hundreds and thousands of such puzzle pieces in the literature, each documenting single witnesses of our becoming, but we rarely look at the bigger picture. 

## 2. Materials and Methods

Hard facts about cranial capacity come from fossils that preserve enough of the braincase to reconstruct its size and shape or represent sediments filling the cranial cavity that hardened over time and are referred to as “endocasts”. Cranial capacity (CC) data and the chronological age (CA) of all hominin fossils were obtained from published scientific sources. Some 193 specimens from the Late Miocene to the Late Pleistocene (thus between 7.3 million years and 10,000 years ago) could be included (Table S1; for references of sources see Supplementary Information). All juvenile specimens with immature brain development (corresponding to an individual age of <11 years in modern humans) were excluded (e.g., Taung 1, A.L. 333-105, KNM-WT 15000, KNM-ER 42700, Engis 2, Gibraltar 2, La Quina 18, Skhul 1). Subadult specimens comparable to ≥11 years were kept in the analyses because brain volume had reached >95% of adult size in extant humans [24]. For their particular position as side branches in the hominin tree (see Figure S1), *H. floresiensis* (insular dwarfism) and *H. naledi* (a Late Mid-Pleistocene primitive hominin featuring adaptations to arboreal life and other Australopithecine-like traits, with no tool industry associated) were not considered in the analysis. However, to check possible effects, computations were repeated including these specimens. The results were only very slightly different and would not change any of the conclusions. 

Inevitably, there are ambiguous data for both CC and CA in the literature. Data were cross-checked, and an emphasis was given to the latest results in the published record, using more modern methods for both CC measurements/estimates and CA dating. 

**Figure 1:**
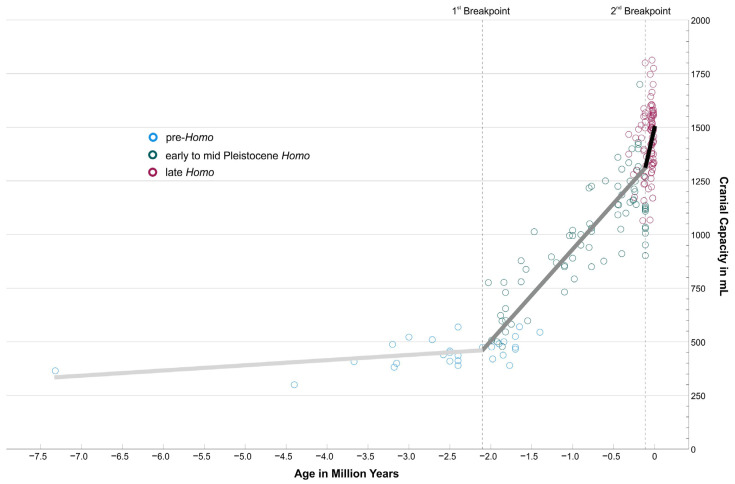
Cranial capacity in mL of 193 hominin fossils from the Late Miocene to the Late Pleistocene. Specimens were coloured according to their taxonomic affiliation (pre-*Homo* blue; Early to Mid-Pleistocene *Homo* green; late *Homo* wine red). For the segmented regression, taxonomy was ignored; only cranial capacity and age were considered. Breakpoints were detected at −2.104 mya and −0.113 mya, respectively. The corresponding regression segments are shown in the graph (1st segment in light gray, y = 512.08 + 24.93x; 2nd segment in middle gray, y = 1365.93 + 430.83x; 3rd segment in black, y = 1516.60 + 1764.83x).

CC was plotted against CA. To find a statistical model providing calculated estimates for the time of the two breakpoints, which are visually obvious in the scatterplot (Figure 1), a segmented regression (also called a broken stick regression or piecewise regression) was used. It estimates break points and coefficients using an iterative fitting of linear models. The R package “segmented” (version 1.6-2) in version R 4.2.2. (© The R Foundation) [25,26] was applied with no a priori specification where breakpoints would be assumed and with no information about the taxonomy used.

In order to add more general knowledge from the fossil record, besides mere CC and CA data, specimens were also grouped according to their published taxonomic affiliation, which includes an assessment of the individual morphologies (which goes far beyond CC). Without entertaining longstanding disputes about the exact classification, three groups, representing relatively unambiguous larger clusters, were used:

1. **Pre-*Homo*:** early hominins, Australopithecines, Paranthropines;
2. **Early to Mid-Pleistocene *Homo*:** Habilines, Erectines, Mid-Pleistocene *Homo*;
3. **Late *Homo*:** Neanderthals, *Homo sapiens.*

Although this grouping is based on a conservative and broad taxonomy, all three groups correspond roughly to the evolutionary stages defined in the discussion. For statistical analyses, IBM SPSS Statistics V28 was used. A k-means cluster analysis was performed setting the number of groups to three. Although only CC and CA were used as input variables, the analysis agreed with the above grouping in 79.3% of cases, which shows that the division makes sense in the context of the present analysis. 

## 3. Results

Data of 193 fossil specimens from the Late Miocene to the Late Pleistocene were re-evaluated with regard to current cranial capacity estimates and dating (see Section 2 and Supplementary Information). As Figure 1 shows, the increase is far from being linear over the observation period from 7.3 million years to 10,000 years ago. This general observation was also found by other authors based on other or more slim versions of cranial capacity databases than those used here [19,20]. Instead, there is only a very slow increase recognizable until roughly two million years ago (mya), when cranial capacities remained below or slightly above 500 mL (blue circles in Figure 1), only mildly above what the great apes feature today. This flat trajectory is even continued until 1.4 mya by Paranthropines (see Figure S1 in Supplementary Information), who invested into a development towards massive mastication apparatuses and low-grade diets. In contrast, *Homo* after 2 mya took another pathway (green circles in Figure 1), investing into larger brain volumes (and reduced masticatory features). Approximately 300–200 thousand years ago (kya), Neanderthals (with some earlier exceptions) and *Homo sapiens* started to roam the Old World (wine-red circles in Figure 1). 

Table 1 summarizes the most important statistical results. Pre-*Homo*, represented by early hominins, Australopithecines, and Paranthropines, shows an average cranial capacity of 453.93 mL ± 63.72 mL (±1 S.D.) Early to Mid-Pleistocene *Homo*, represented by Habilines, Erectines, and Mid-Pleistocene *Homo*, feature a much higher, more than doubled, cranial capacity of 1011.97 mL ± 254.54 mL. Late *Homo* (Neanderthals and modern humans) finally show again a significantly increased average cranial capacity and lower variability of 1442.60 mL ± 155.33 mL compared to the former group. Cranial capacities of the three supergroups are significantly different from each other in a Kruskal–Wallis Test (see also Boxplot in Figure S2 Supplementary Information). The Spearman correlation between cranial capacity and chronological age is r_s_ = 0.837, meaning that ~70% of the total variation of cranial capacity can be explained by chronological age. 

The segmented regression, which ignores the grouping and only uses the metrics “Cranial Capacity” and “Chronological Age”, finds two breakpoints at −2.104 mya (±1 S.E. −2.251 mya to −1.957 mya) and at −0.113 mya (±1 S.E. −0.177 mya to −0.049 mya) (Table 1). The regression segments are plotted in Figure 1. The data are consistent with two strong changes in the increase rate of cranial capacity, at ~2.1 mya and ~113 ka. The roughly corresponding overlap with the three supergroups is remarkable. 

These results document clearly the differences in cranial capacity between pre-*Homo*, Early to Mid-Pleistocene *Homo*, and late *Homo*, the progressive association between cranial capacity and chronological age, and the nonlinear development of hominin cranial capacity from the Late Miocene to the end of the Pleistocene. The following discussion will put these data into context with our fossil and archaeological record to illuminate the quantum leaps in our evolution and their connection to brain development.

## 4. Discussion

### 4.1. A Slow Start before the First Quantum Leap

It is safe to assume that the forerunners of humans were subject to the same evolutionary forces as all other living beings for millions of years. This leans on the ideas that Darwin and many others developed and described over the last 200 years, and which we can find in the textbooks. In an analogy in modern language usage, we could call it “Evolution 1.0”. This means basically that random changes in the genome (e.g., through mutation and recombination) and natural selection are main factors in triggering adaptations of populations to changing environments. Additional random factors such as gene drift, founder effects, and others affect the fate of a population. The overproduction of individuals and their diversity are the substrate for selection, which implies unequal reproduction among members. In this regard, the very first hominins and the successive Australopithecines during the Late Miocene and the Pliocene were just as exposed to these natural forces as the famous Darwin finches on the Galapagos Islands, and probably also the earliest representatives of our *Homo* lineage, which tentatively emerged between 2.8 and 2.5 million years ago [27]. Fossil remains and artefacts are notoriously sparse at the transition from Australopithecines to *Homo*, and dating issues leave us with further uncertainties. Simple stone tools were already in use in several African locations in those times, and they were not necessarily associated with the genus *Homo* [28]. The Lomekwian and Oldowan industries between 3.3 and 1.7 million years ago are the first preserved evidences of this tool technology. Those tools were usually made by chipping off one flake, or a few flakes, of stone with another stone, but show no greater sophistication. Their production is straightforward and can be quickly acquired [29]. These tools appear hundreds of thousands of years before any evidence of brain expansion [30]. Tools were becoming more frequent and widespread approximately two million years ago when brain and body size showed evidence of increase [31]. This also fits the first breakpoint found in the present analysis of cranial capacity (Figure 1).

Approximately 1.8–1.7 million years ago, a watershed moment emerged in our history: a new and much advanced tool culture, the Acheulean, appeared. Its typical hand axes are worked symmetrically and on both sides (they are iconic of the Stone Age in popular displays). Untrained modern humans struggle with the demanding and complex technique, and guidance without explicit verbal instructions is difficult [32]. The ability for such complex stone knapping was connected with the development of specific brain regions that show differences between macaques, chimpanzees, and humans, and particularly relate to the “how” in executing or copying bodily actions [33]. 

Fire is much less likely to feature than hard evidence such as stone tools, thus, the proof of anthropogenic fire is difficult. Nevertheless, some sites in East Africa point at least to the opportunistic use of fire between 1.6 and 1.5 million years ago [34]. Since 1.8 million years ago, we also note in the fossil and archaeological record that *Homo* (represented by species such as *H. erectus*, *H. ergaster*, or *H. georgicus*, a taxonomic labelling that is secondary to our considerations here) is the first hominin to leave Africa [35]. *Homo*’s body height and proportions, with long legs and short arms, are already typical for modern people [36]. Australopithecines were predominantly bipedal, but their relatively long arms and curved finger bones indicate a still pronounced adaptation to tree life. *Homo*, in contrast, seems to use a different strategy, showing a significant increase in brain size, particularly after two million years BP (before present, Figure 1), in concert with internal brain reorganization [20,37]. The evolution of enhanced vocal communication was suggested to fall also into the times of *Homo erectus* [38]. Reliance on stone-tool making may have generated a selection for teaching and language [39]. The same authors suggest that the long stasis for approximately 700,000 years during the Oldowan could be explained by low-fidelity social transmission, while the later developing teaching and protolanguage may have been prerequisites for the appearance of Acheulean industry. All the innovations mentioned above suggest an important milestone starting to occupy a “dedicated cognitive niche” [40]. This departure from the canonical pathway of other animals’ evolution characterizes the beginning of “Evolution 2.0”.

*Homo* was physically a relatively weak creature compared to other predators and food competitors. *Homo* had no fangs or claws and was a comparatively slow runner and a poor climber. Utilizing intellectual and social skills, humans nevertheless succeeded in expanding their range from Africa into large parts of Eurasia during the Early Pleistocene. The inclusion of abiotic expedients such as more sophisticated stone tools, fire, and weapons increased further over time, and the pronounced climatic fluctuations and falling temperatures in the Middle Pleistocene could not endanger *Homo*’s existence even in the northern hemisphere. Still, if clothing or footwear have not been preserved during this period, we can assume that *Homo* also used these aids to compensate for the disadvantages of a reduced hair coat. Approximately 300,000 years ago (300 ka), the archaeological record at Schoeningen (Germany) documents wooden spears [41], a first evidence for thoughtfully produced distance weapons. At approximately the same time, the ubiquitous and constant use of fire is noted [42,43]. At approximately 200 ka, new progressive tool cultures using prepared-core techniques (Middle Paleolithic, Middle Stone Age), which are different from the preceding Acheulean, appeared. The gradual advancements through the Middle Pleistocene give proof of the increasing alleviation of natural pressures on humans and the refinement of technology.

### 4.2. The Second Quantum Leap and the Emergence of Symbolic Creatures

Eventually, we see a change in the increase rate of cranial capacity approximately 113 ka (second breakpoint in Figure 1). While there existed pre- and early Neanderthals earlier, the fossil record increases considerably from 130 ka onwards, particularly with the later “classic” Neanderthals from the European Würm (115 ka—Holocene). However, Neanderthal-like morphology was not bound solely to Europe; West and East Asian fossils also show typical Neanderthal features to various degrees [44]. On the other hand, modern humans had already left Africa by 185 ka [45] and started to spread across the Old World. The earliest stages of the *Homo sapiens* clade so far are documented from Jebel Irhoud (Morocco), dated to approximately 315 ka [46]. The fossils feature short faces, small teeth, and reduced brow ridges compared to other Mid-Pleistocene specimens. These traits were interpreted as evidence of a domestication syndrome, suggestive of the fact that modern humans evolved greater docility than their pre-*Homo sapiens* ancestors [47].

Domesticated animals tend to show a suite of common anatomical and behavioral characteristics, a fact already recognized by Darwin [48]. Working with silver foxes and minks, Belyaev [49], a hundred years later, revealed that features of the domestication syndrome were produced by selection purely for docility. Recently, similar changes could also be found for non-mammalians, in that case, for chickens [50]. 

Several studies suggest that *Homo sapiens*, but not Neanderthals, had a domestic syndrome (e.g., [47,51,52,53]). Anatomical signs are a reduction in body mass, a shortening of the face and a reduction in tooth size, reduced sexual dimorphism (feminization), and a reduction in cranial capacity. All of them apply to modern humans, although there is some ambiguity with regard to the last one. The absolute average cranial capacity of early (200–76 ka) Neanderthals is significantly lower than in contemporary modern humans; the cranial capacity of classic (75–27 ka) Neanderthals is approximately equal to that of contemporary modern humans [54]. Another study confirms that Neanderthal brains were in the upper size variation of modern humans, but not larger [55]. However, early modern humans (200–76 ka) show a higher endocranial volume than later ones (75–27 ka) [54], thus, within the modern human lineage, a modest decrease from 1535.50 to 1473.84 was recognizable in this study (see also [56] p.193 for Holocene *Homo sapiens*). Additionally, facial width and brow ridge projection have declined in *Homo sapiens* [57]. The 2D/4D ratio of modern humans, the relative length of the second to the fourth finger, is suggestive of low prenatal androgen exposure in utero [58]. Neanderthals show a more masculinized 2D/4D pattern [52], and together with their relatively robust overall anatomy, this suggests that the gracilization and neotenization of modern humans happened after the split of the two lineages. 

Compared to our closest living relatives—wild chimpanzees and bonobos—humans show far lower frequencies of within-group aggressive conflicts [59]. Adult male humans with relatively narrow faces were also found to be less reactively aggressive, and are perceived as being less aggressive [60,61]. Bonobos show a similar pattern, being less aggressive than common chimpanzees, and featuring traits of domesticates such as juvenilized patterns of development [62]. A selection for low propensity for reactive aggression, in connection with larger group sizes and gaining easier access to within-group leadership via coalitions rather than using fighting prowess, could be responsible for the occurrence of the domestication syndrome in modern humans. There are no simple explanations of which evolutionary mechanisms might have led to the self-domestication of modern humans, e.g., genetic group selection, group-structured culture selection, female mate choice, self-control, etc. [47]. Nevertheless, using established relationships between brain and bonded group size across anthropoid primates, the hypothetical group size for early modern humans was shown to be larger than that for Neanderthals [54]. Enhanced social complexity goes along with larger group size, which itself requires a larger number of relationships to maintain and opens a wider range of coalitions that can potentially be built. It seems that modern humans were better adapted for larger social networks, while Neanderthals kept physical robustness and increased the size of their eyeballs [54]. 

Overall, cranial capacity data show that, at approximately 113 ka, there is again a change in the increase rate; brains were growing bigger faster (Figure 1). Data also demonstrate that late *Homo* showed an average cranial capacity that is 42% larger than Early to Mid-Pleistocene *Homo*, even if modern humans experienced a modest decrease in their later evolutionary development, which might be due to a domestication syndrome. Modifications of cognitive abilities are not only a consequence of enlarged brain volume but also cortical reorganization; these are related to each other [63]. In particular, the relatively enlarged parietal lobe together with the “globularization” of the braincase could be considered derived features of modern humans [37]. It is noteworthy that Neanderthals and modern humans have the largest cranial capacities but show different relative contributions of the frontal, parieto-temporal, and occipital lobes to the brain surface [37]. While the Jebel Irhoud specimens showed *Homo sapiens* faces [46], the shape of their braincase was still elongated. As in Neanderthals, the braincase had not yet arrived at the highly bulging, rounded cranial vault that is so typical of modern humans [21,56]. Nevertheless, annular constructions at Bruniquel Cave (France) dated to 176 ka document one of the first edifices, which are attributed to early Neanderthals [64]. Some time later, the archaeological record surprises us with something new—the first hints of ornaments produced by humans (130 ka in [65]; 115 ka in [66]). The “globularization” of the human brain can only be recognized at approximately 100 ka in modern humans [21], but not in contemporaneous Neanderthals. They seemed to have large brains but retained the genes responsible for the retention of archaic elongated braincases [67]. 

Tangible evidence of symbolic behavior appears at approximately 73 ka at Blombos Cave in South Africa in the form of an ochre drawing on a silcrete flake, which represents one of the first abstract and figurative drawings [68]. Eggshell engravings from Diepkloof fall into a similar time range, but there is discussion whether all these representations were only decorations or were linked to semantic content [69].

**Figure 2:**
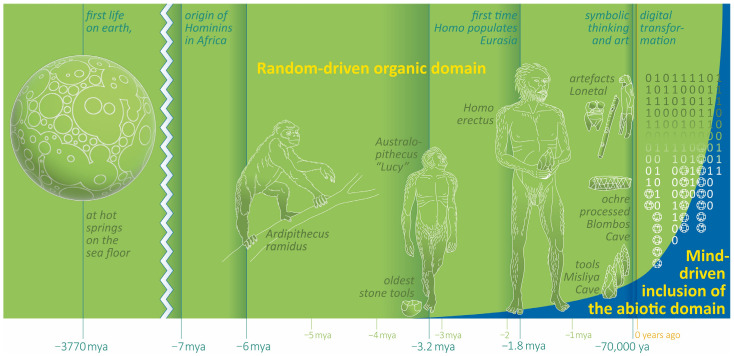
Milestones of human evolution and the progressive inclusion of the abiotic domain. A principle scheme.

In Spain, a hand stencil and red wall paintings attest the impulse of humans to eternalize themselves on a cave wall approximately 64 ka [70]. The claim that these traces originate from Neanderthals is contested [71]. However, another indirect proof of Neanderthal symbolic capabilities comes from Einhornhöhle (Germany): a 51 ka engraved giant deer phalanx [72]. Modern humans might already have been in Europe since 54 ka [73]. No doubt at all exists that, with the arrival of modern humans, the Eurasian continent witnessed a flood of new artefacts, consisting of musical instruments and figurines (dated at Geißenklösterle to 42 ka [74]), murals (Lubang Jeriji Saléh >40 ka [75]), and a multitude of personal ornaments and widely refined and diversified tools (Aurignacian [74]). All these innovations document the progressive cognitive investment and inclusion of abiotic elements into people’s lives (Figure 2). To a significant extent, these objects are not required for mere survival. However, they testify to a proliferation in mental abilities and social structure, and, at least at this point, they strongly suggest the existence of an abstract language. Progressing with symbolic storage, humans had made an intellectual quantum leap heralding the next stage in our evolution—“Evolution 3.0”. 

Symbolic communication promotes logical thinking and enables ideas and conceptions about the world to be transmitted through time. The only storage of symbolic information until then were the brains of the survivors, which is one reason, for instance, why older clan members were valuable to society. Humans began to materialize their thoughts and concepts in multiple forms long before they settled down. During the expiring Pleistocene, the archaeological record of artefacts increases in quantity and quality. Finally, after the agricultural revolution of the Holocene (12.5 ka), imposing buildings (e.g., Göbekli Tepe 11 ka, Ġgantija 5.7 ka, Knap of Howar 5.6 ka, Stonehenge 5 ka, Gizeh 4.5 ka, and Nuraghe 3.6 ka), writings (5.5 ka), countless works in the performing arts and literature, scientific treatises, money, and hierarchically organized empires with growing populations emerged. Humans started to intervene dramatically with the ecology of the ecosystems, an activity that has continued until today.

### 4.3. The Final Quantum Leap: Intentional Evolution

Symbolic language and the increasing complexity of tools and social relationships were necessarily coupled with increasing individual time and effort to adapt successfully to a more demanding environment. Learning new behaviors (an ontogenetic adaptation) may positively affect reproductive success and thus over time may have effects on the genetic makeup of the group through natural selection (thus resulting in a phylogenetic adaptation [76]). This phenomenon has been discussed since the end of the 19th century as the “Baldwin effect” [77], but could rarely be demonstrated in experiments (productively, e.g., for *Drosophila melanogaster* [78]). However, genetically inherited lactase persistence in response to pastoral behavior, for instance, is discussed as an example. Raising domesticated animals, some individuals would have been able to digest animal milk. This additional resource had a high rate of renewal, included essential nutrients, and delivered less contaminated liquid than water. This might have helped to increase the fitness of those individuals exploiting animals’ milk. Over time, those genetic variants tolerable to lactose eventually became more frequent in the population, and, finally, the ability to digest dairy products could have been established in the gene pool. 

The idea of the Baldwin effect, a fitness-based argument, is that individual adaptations to new environmental conditions may allow a population to survive while providing more time for genetic evolution to “catch up” ([79]; see also the demarcation between Baldwin effect and genetic assimilation, a developmental-based argument). Without doubt, the Baldwin effect needs to be considered as an important factor for a species such as humans, whose evolution is significantly dependent on social learning and cultural transmission as a second inheritance system. Adopting new behaviors to survive and exploit new habitats and ecological niches is an outstanding quality of the genus *Homo*. While niche construction had considerable selective effects on human populations at all times, it is reaching an unprecedented scale now (not just with regard to climate). Some areas of the planet might not remain habitable, or be habitable only at great expense. Some previously favorable traits such as physical strength, running speed, or dexterity will lose, or have already lost, their selective advantages. Gender roles in societies are changing, which means that females are gaining more access to decision making, finally extending their societal impact far beyond reproductive capabilities. Underrepresented minorities are noticed to a larger extent, at least in some industrialized societies. These transformations mean that females and males who are better adapted to the new inclusive behavior could be more successful in the sense of the Baldwin effect. 

Humans are the first creatures on Earth with the capacity to interfere directly with the fundaments of biology instead of waiting for the relatively slow natural forces that transform the gene pool of a species. The term is not yet used in our language, but it is tempting to refer to the consequences of this capacity as “intentional evolution”, although not all consequences will be intentional and appreciated. The ability to consciously intervene with evolutionary processes, an “Evolution 4.0”, is accompanied by a yet unmatched maximum dissociation from natural environments. Most average citizens of industrialized countries are surrounded more by abiotic resources than by nature. They live and work in houses, move mainly using cars, bicycles, trains, and planes, and spend considerable free and working time in virtual worlds, which are projected on screens. Basal functions of the human neocortex such as memory or calculations are partially or almost completely outsourced to machines, intelligence is being created artificially, social media produce a superstructure in the post-private era made up of billions of complex brains, and technology allows us to directly intrude into the genome and proteome. Traits such as tolerance for monotony or loneliness could become important dispositions in modern societies.

Most actions so far have been typically driven by individual activities on the part of politics, economy, and science. Probably only the toughest conspiracists could detect a greater master plan or coordination behind our staggering path towards transhumanism [80]. Indeed, we are moving forward without much reflection, not intentionally. One example is that female academics in some industrialized countries remain 18–26% childless [81]. If an academic degree had something to do with brain performance, this would be a selection in a disadvantageous direction. Likewise, the artificial control of a pathogen prevents any genetic immunity against the pathogen from being selected. On the other hand, modern medicine, a learned behavior that generally improves fitness, prevents genetic adaptation. The COVID-19 pandemic has recently taught us that we can only escape from some threatening conditions if we move on faster than nature with our technological innovations. 

The difference between affecting our evolution rather randomly as a byproduct of other aims on the one hand and “intentional evolution” on the other hand needs some clarification here. Mate choice is one of the primary mechanisms of evolution. Indeed, it affects the evolution of a species fundamentally, but it also works for the zebrafish without the fish knowing anything about evolution. What about humans who started thinking consciously about reproduction? Human-made selection was applied to crops and animals for thousands of years, and not only members of dynasties were long aware of the importance of their offspring’s mate selection for future generations. This actually also represents a planned intervention into the gene pool of a population, but, until quite recently, just without knowing anything about genes or evolution. What is meant here by “intentional evolution” is a direct, technology-driven, deliberate intervention into the gene pool with the goal of altering it. 

The obstetrical dilemma was appreciably reduced by the cesarean section [82], which alleviates the selective pressure on birth canal dimensions and the infant’s head circumference. Although it would change the gene pool, this was not the primary intention of this intervention; it is somehow in a gray zone. In contrast, a fetus with gene defects is now diagnosable, and pregnancy can be terminated. This is a direct intervention into the gene pool at an advanced stage of the embryonic development. Reproductive medicine deals with the selection of gene-defect-free embryos in the test tube, which are then successfully implanted—another direct intervention into the gene pool, this time at an early stage of embryonic development. The case of He Jiankui [83] using genome editing to disable an unwanted gene shows where more direct interventions would lead to if permitted. With CRISPR/Cas9 and other developments, completely new properties could be created, and unwanted genes and diseases could be eliminated. People of smaller stature, for instance, would need less energy, or artificially induced meat allergies could help in solving the carbon dioxide problem. At the moment, factors such as beauty, intelligence, disease resistance, tolerance to pollutants, or electromagnetic radiation are not yet artificially selected (but are partly influenced by mate choice) or created in a lab because of ethical considerations, but the technology is in reach. Nanotechnology provides us with tools to tinker with human anatomy and physiology at the cellular level and to restore or improve functions. This is not directly interfering with the genome, but it might also have long-term effects with regard to natural selection.

### 4.4. Technological Posthumanism?

Recapitulating our history of the last million years (Figure 2), we recognize that human biocultural evolution is closely coupled with two trends:

(i) Employing sophisticated cognitive strategies and the utilization of abiotic resources became the prevailing principle;
(ii) This development accelerates over time.

In his famous book *The Singularity Is Near*, Raymond Kurzweil [23] deconstructed “evolution” as a whole into six epochs, which he designed as much larger units than the stages of human evolution in this current contribution here. Instead of focusing on the evolution of the genus *Homo*, he refers to the development of the entire universe. Kurzweil’s epochs start with “Physics and Chemistry” and end with the conquest of the whole universe. However, the idea of leaps from one stage to the other, entailing entirely new opportunities, is similar. Kurzweil sees the exponential growth of computing power flowing into a singularity—basically, an irreversible turning point. While his calculations for the date of occurrence are meanwhile obsolete (we are, for instance, still quite far from understanding the human brain, let alone simulating all its functions), the question remains: what would happen if machines could simulate our way of thinking? Machines can run all day at full power, and they could work independently from us and optimize themselves much faster than we could do. 

Other masterminds such as Stephen Hawking [84], Elon Musk [85], and many more have speculated in the last decade about advances in artificial intelligence (AI) and the consequences for humankind; this, of course, remains a purely heuristic endeavor. Nevertheless, “Evolution 5.0”—the complete displacement of biological humans or the post-human era—should not be left out of the considerations of the evolutionary anthropologist. The exponential growth of our brains is well visible in Figure 1, and the question is now obviously how we should include the cognitive aids we are currently using—such as computers—into the graph of cranial capacity for the last half century and the time following. Cranial capacity serves as a proxy for cognitive performance, but this does not consider any tools that help increase calculation or memory power. The rapidly growing inclusion of abiotic means into our lives, captured in Figure 2, is also obvious. In the preceding chapter, we saw that a hybridization of humans and machines is already underway in terms of intellectual capacities. There are also approaches visible on the physical level, especially in the area of prosthetics, for instance, experimenting with computer-controlled limbs. Such a robotic arm can develop much more power than a human arm, which could make cyborgs more attractive than biotic humans, for instance, for military purposes. 

The next logical step, as Kurzweil and others anticipated, is the development of abiotic creatures that are equipped with AI to improve and reproduce independently. After Pandora’s box has been opened, humans could be replaced by abiotic beings. This is not necessarily an implausible cyber-romantic vision [86]. Creative Machines Lab at Columbia University [87] is currently designing machines that can design and build other machines. A female-looking robot with empathic abilities named “Sophia”, developed by Hanson Robotics in Hong Kong, was actually declared a real citizen of Saudi Arabia in 2017 [88]. 

The European Union identifies that a major issue in the context of the digital transformation is “… to ensure Artificial Intelligence is developed in ways that respect people’s rights and earn their trust”. (https://ec.europa.eu/info/strategy/priorities-2019-2024/europe-fit-digital-age/shaping-europe-digital-future_en (accessed on 22 March 2023)). The U.S. Department of State indicates that “… advances in AI technology present both great opportunities and challenges” (https://www.state.gov/artificial-intelligence/ (accessed on 22 March 2023)). The OECD’s recommendation on artificial intelligence (https://legalinstruments.oecd.org/en/instruments/OECD-LEGAL-0449 (accessed on 22 March 2023)) clearly points out the role of the needed trustworthiness of the new technology. 

Human biocultural evolution is shifting from a randomly driven natural process to a mind-driven transformation guided by humans. This is a type of responsibility for which we have not yet developed mechanisms. Most documents from the large governmental agencies (e.g., EU, US, OECD) do not even acknowledge any effects on the biology of populations, but we are becoming able to interfere directly with our genome, epigenome, and proteome. It is probably time to intensify this discussion now.

## Figures and Tables

**Table 1:** Statistical results.

*Cranial Capacity in mL*	N	Mean	Std. Deviation
*Pre-Homo*	28	453.93	63.72
*Early to mid Pleistocene Homo*	76	1011.97	254.54
*Late Homo*	89	1442.60	155.33
***Kruskal-Wallis Test***	**H**	**df**	**Asymp. Sig.**
	138.14	2	<0.001
***Spearman Correlation***	**Coefficient**	**B**	**Sig. (2-tailed)**
*Cranial Capacity x*	0.837	0.701	<0.001
*Chronological Age*			
***Segmented Regression***	**Breakpoint**	**Estimate**	**Std. Error**
*Cranial Capacity x*	1st	−2.104 mya	±0.147 mya
*Chronological Age*	2nd	−0.113 mya	±0.064 mya
	Adjusted R^2^ = 0.8494, *p* < 0.001		

## Data Availability

This contribution is based on published results from numerous sources which are publicly available and all listed in Table S1.

## Supplementary Materials

The following supporting information can be downloaded at: https://www.mdpi.com/article/10.3390/life13041030/s1. Table S1: Specimens in the study, cranial capacity, chronological age, taxonomy, and data sources; Figure S1: Cranial capacity in mL of 193 hominin fossils from the Late Miocene to the Late Pleistocene; Figure S2: Boxplot of cranial capacities for the three hominin super-groups.
