# Supplementary material for: Quantum Leaps in Human Biocultural Evolution and the Relationship to Cranial Capacity
Source: Life (Basel). 2023 Apr 17;13(4):1030. doi: 10.3390/life13041030 (PMC10145355; doi:10.3390/life13041030)
Supplement: Supplementary file 1 [file life-13-01030-s001.zip › life-2230172-supplementary.pdf]

Supplementary Information to Weber, G.W.

Quantum Leaps in Human Biocultural Evolution and the Relationship to Cranial Capacity

Author: Gerhard W. Weber
Affiliations: [1] Department of Evolutionary Anthropology, University of Vienna, Austria
[2] Human Evolution and Archaeological Sciences (HEAS), University of Vienna, Austria

Corresponding author: Gerhard W. Weber
Department of Evolutionary Anthropology
University of Vienna
Djerassiplatz 1
A-1030 Vienna, Austria
gerhard.weber@univie.ac.at

Table S1: 193 specimens in the study; cranial capacity (CC in milliliters), chronological age (CA in million years), taxonomy, and data sources.

Specimen	CC in mL	CA in mya	Taxonomy	Source
Abri Pataud 1	1323	0,027	Homo sapiens	All: Neubauer et al., 2018
AL 162-28	400	3,150	Australopithecines	All: Shultz et al., 2012
AL 288-1 ("Lucy")	382	3,180	Australopithecines	Endocranial Volume & Taxon: Gunz et al., 2020, Age & Taxon: Schwartz & Tattersall, 2005, page 51
AL 333-45	488	3,200	Australopithecines	Endocranial Volume & Taxon: Gunz et al., 2020, Age & Taxon: Schwartz & Tattersall, 2005, page 51
AL 444-2	522	3,000	Australopithecines	Endocranial Volume & Taxon: Gunz et al., 2020, Age & Taxon: Schwartz & Tattersall, 2005, page 51
Amud 1	1747	0,053	Neanderthals	All: Neubauer et al., 2018
Arago 21	1138	0,438	Mid-Pleistocene Homo	Age: Falguères et al., 2015, Endocranial Volume & Taxon: Shultz et al, 2012
ARA-VP-6/500 ("Ardi")	300	4,400	Early Hominins	Age & Taxon: Renne et al., 1999, Endocranial Volume & Taxon: Suwa et al., 2009
Atapuerca 4 (SH 4)	1360	0,448	Mid-Pleistocene Homo	Age & Taxon: Demuro et al., 2019, Endocranial Volume& Taxon: Poza-Rey et al., 2019
Atapuerca 5 (SH 5)	1092	0,448	Mid-Pleistocene Homo	Age & Taxon: Demuro et al., 2019, Endocranial Volume& Taxon: Poza-Rey et al., 2019
Atapuerca 6 (SH 6)	1225	0,448	Mid-Pleistocene Homo	Age & Taxon: Demuro et al., 2019, Endocranial Volume& Taxon: Poza-Rey et al., 2019
Biache 1 (Biache-Saint-Vaast 1)	1200	0,240	Mid-Pleistocene Homo	Age: Bahain et al., 2015, Endocranial Volume: Shultz et al., 2012, Taxon: Guipert et al., 2011
Bodo	1250	0,600	Mid-Pleistocene Homo	All: Shultz et al., 2012
Border Cave 1	1510	0,164	Homo sapiens	All: Shultz et al., 2012
Bouri (Bou-VP-12/130)	450	2,500	Australopithecines	All: Shultz et al., 2012
Brno I	1600	0,026	Homo sapiens	All: Shultz et al., 2012
Brno II	1500	0,029	Homo sapiens	Age: Kuzmin & Keates, 2014, Endocranial Volume & Taxon: Shultz et al., 2012
Brno III	1304	0,026	Homo sapiens	Age & Endocranial Volume: Shultz et al., 2012, Taxon: Schwartz, 2002, page 81
Bruniquel 2	1555	0,012	Homo sapiens	All: Shultz et al., 2012
BSN12/P1 (Gona)	896	1,260	Erectines	All: Baab et al. 2022
Buia (UA 31)	995	1,000	Erectines	Age: Ghinassi et al., 2015, Endocranial Volume & Taxon: Bruner et al., 2016
Cap Blanc 1 ("Magdalenian girl")	1434	0,012	Homo sapiens	All: Shultz et al., 2012
Ceprano	1185	0,400	Mid-Pleistocene Homo	All: Shultz et al., 2012
Chancelade	1700	0,018	Homo sapiens	Age & Taxon: Barshay-Szmidt et al., 2016, Endocranial Volume & Taxon: Shultz et al., 2012
Cioclovina	1499	0,033	Homo sapiens	All: Kraniotii et al. 2011
Combe Capelle	1457	0,010	Homo sapiens	Endocranial Volume & Taxon: Neubauer et al., 2018, Ae & Taxon: Hoffmann et al., 2011
Cro-Magnon 1	1574	0,031	Homo sapiens	All: Neubauer et al., 2018
Cro-Magnon 3	1813	0,031	Homo sapiens	All: Neubauer et al., 2018
Daka (BOU-VP-2/66)	995	1,040	Erectines	All: Shultz et al., 2012
Dali 1	1160	0,263	Mid-Pleistocene Homo	Age: Sun et al., 2017, Taxon & Endocranial Volume: Shultz et al., 2012
DAN5/P1 (Gona)	598	1,550	Erectines	All: Baab et al. 2022
Dmanisi D2280 (Skull 1)	730	1,820	Erectines	Endocranial Volume: Rightmire et al., 2017, Age: Ferring et al., 2011, Taxon: Lordkipanidze et al., 2013
Dmanisi D2282 (Skull 2)	655	1,820	Erectines	Endocranial Volume: Rightmire et al., 2017, Age: Ferring et al., 2011, Taxon: Lordkipanidze et al., 2013
Dmanisi D2700 (Skull 3)	600	1,820	Erectines	Endocranial Volume: Rightmire et al., 2017, Age: Ferring et al., 2011, Taxon: Lordkipanidze et al., 2013
Dmanisi D4500 (Skull 5)	546	1,820	Erectines	Endocranial Volume: Rightmire et al., 2017, Age: Ferring et al., 2011, Taxon: Lordkipanidze et al., 2013
Dolni Vestonice 14	1663	0,031	Homo sapiens	Age: Fewlass et al., 2019, Endocranial Volume & Taxon: Neubauer et al., 2018
Dolni Vestonice 18	1481	0,031	Homo sapiens	Age: Fewlass et al., 2019, Endocranial Volume & Taxon: Shultz et al., 2012

Dolni Vestonice 20	1378	0,031	Homo sapiens	Age: Fewlass et al., 2019, Endocranial Volume & Taxon: Shultz et al., 2012
Dolni Vestonice 21	1547	0,031	Homo sapiens	Age: Fewlass et al., 2019, Endocranial Volume & Taxon: Shultz et al., 2012
Dolni Vestonice 3	1322	0,031	Homo sapiens	Age: Fewlass et al., 2019, Endocranial Volume & Taxon: Shultz et al., 2012
Ehringsdorf 9	1450	0,230	Neanderthals	All: Holloway, Broadfield, & Yuan, 2004, page 298
Eyasi 1	1235	0,130	Homo sapiens	All: Shultz et al., 2012
Feldhofer 1 (Neanderthal 1)	1261	0,043	Neanderthals	Endocranial Volume & Taxon: Neubauer et al., 2018, Age & Taxon: Kuzmin & Keates, 2014
Fish Hoek 1 (SAM-AP4692)	1335	0.007	Homo sapiens	CC: Keen 1942, Age: Stynder et al. 2009
Florisbad 1	1280	0,259	Homo sapiens	All: Shultz et al., 2012
Fontéchevade 2	1420	0,046	Homo sapiens	All: Shultz et al., 2012
Galilee (Zuttiyeh)	1400	0,200	Mid-Pleistocene Homo	All: Shultz et al., 2012
Ganovce 1	1320	0,107	Neanderthals	All: Shultz et al., 2012
Gibraltar 1 (Forbe's)	1213	0,075	Neanderthals	All: Neubauer et al., 2018
Grotte des Enfants 4	1775	0,013	Homo sapiens	Age & Taxon: Kuzmin & Keates, 2014, Endocranial Volume & Taxon: Shultz et al., 2012
Grotte des Enfants 5	1375	0,013	Homo sapiens	Age & Taxon: Kuzmin & Keates, 2014, Endocranial Volume & Taxon: Shultz et al., 2012
Grotte des Enfants 6	1580	0,013	Homo sapiens	Age & Taxon: Kuzmin & Keates, 2014, Endocranial Volume & Taxon: Shultz et al., 2012
Harbin (H. Longi)	1420	0,200	Mid-Pleistocene Homo	CC: Ji et al. 2021; Age: Shao et al. 2021
Herto (BOU-VP-1/16)	1450	0,160	Homo sapiens	All: Shultz et al., 2012
Hexian (PA 830)	1025	0,412	Erectines	All: Shultz et al., 2012
Hofmeyr	1541	0,036	Homo sapiens	All: Neubauer 2022
Hualongdong 6	1150	0,300	Mid-Pleistocene Homo	All: Wu et al. 2019
Jebel Irhoud 1	1375	0,315	Homo sapiens	All: Neubauer et al. 2018
Jebel Irhoud 2	1467	0,315	Homo sapiens	All: Neubauer et al. 2018
Jinniushan	1317	0,200	Mid-Pleistocene Homo	All: Shultz et al., 2012
Kabwe (Broken Hill)	1249	0,299	Mid-Pleistocene Homo	Endocranial Volume & Taxon: Neubauer et al., 2018, Age & Taxon: Grün et al., 2020
KNM-ER 1470	776	2,030	Habilines	Endocranial Volume & Taxon: Shultz et al, 2012, Age: McDougall et al., 2012
KNM-ER 1805	582	1,750	Habilines	Endocranial Volume & Taxon: Holloway, Broadfield, & Yuan, 2004, page 297, Age: McDougall et al., 2012
KNM-ER 1813	478	1,860	Habilines	Endocranial Volume & Taxon: Benazzi et al., 2013, Age: Feibel et al., 2009
KNM-ER 23000	491	1,900	Paranthropines	Endocranial Volume & Taxon: Brown et al., 1993, Age & Taxon: Schwartz & Tattersall, 2005, page 142
KNM-ER 3732	623	1,880	Habilines	All: Shultz et al., 2012
KNM-ER 3733	878	1,630	Erectines	Endocrancial Volume: Neubauer et al., 2018, Age: Lepre & Kent, 2015, Taxon: Bruner et al., 2015
KNM-ER 3883	838	1,570	Erectines	Endocrancial Volume & Age: Neubauer et al., 2018, Taxon: Bruner et al., 2015
KNM-ER 3884	1400	0,276	Mid-Pleistocene Homo	Taxon & Endocranial Volume: Shultz et al, 2012, Age: Bräuer et al., 1997
KNM-ER 406	525	1,700	Paranthropines	All: Schwartz & Tattersall, 2005, pages 141-142
KNM-ER 407	438	1,850	Paranthropines	All: Falk et al., 2000
KNM-ER 732	466	1,700	Paranthropines	All: Falk et al., 2000
KNM-ES 11693	1212	0,250	Mid-Pleistocene Homo	Age & Taxon: Shultz et al, 2012, Endocranial Volume: Bräuer et al., 2004
KNM-ER 13750	475	1,700	Paranthropines	All: Schwartz & Tattersall, 2005, pages 141-142
KNM-WT 17000	410	2,500	Paranthropines	All: Falk et al., 2000
KNM-WT 17400	390	1,770	Paranthropines	Age & Endocranial Volume: Falk et al., 2000, Age & Taxon: Wood, 2011, page 414
Konso (KGA-10-525)	545	1,400	Paranthropines	All: Holloway, Broadfield, & Yuan, 2004, page 297

Kostenki 14	1222	0,038	Homo sapiens	Age & Taxon: Dinnis et al., 2019, Endocranial Volume & Taxon: Holloway, Broadfield, & Yuan, 2004, page 299
Kostenki 2	1605	0,038	Homo sapiens	All: Holloway, Broadfield, & Yuan, 2004, page 299
Krapina 3 (Cranium C)	1272	0,130	Neanderthals	All: Cofran et al., 2021
Krapina 6 (Cranium E)	1158	0,130	Neanderthals	All: Cofran et al., 2021
Krapina 5 (Cranium D)	1397	0,130	Neanderthals	All: Cofran et al., 2021
La Chaise 1 (Suard 1)	1065	0,143	Neanderthals	All: Shultz et al., 2012
La Chapelle aux Saints	1490	0,052	Neanderthals	All: Neubauer et al., 2018
La Ferrassie 1	1643	0,046	Neanderthals	Endocrancial Volume & Taxon: Neubauer et al., 2018, Age: Guérin et al., 2015
Le Moustier 1	1486	0,041	Neanderthals	Age & Taxon: Holloway, Broadfield, & Yuan, 2004, page 299, Endocranial Volume & Taxon: Shultz et al., 2012
La Quina 5	1345	0,071	Neanderthals	Age & Taxon: Grimaud-Hervé et al., 2020, Endocranial Volume & Taxon: Shultz et al., 2012
Laetoli 18 (LH18)	1237	0,120	Homo sapiens	All: Neubauer et al., 2018
Lantian 1 (Gongwangling 1)	780	1,630	Erectines	Age & Taxon: Zhu et al., 2015, Endocranial Volume & Taxon: Holloway, Broadfield, & Yuan, 2004, page 298
Liujiang	1567	0,115	Homo sapiens	Age & Taxon: Shultz et al., 2012, Endocranial Volume & Taxon: Wu et al., 2008
Maba 1	1300	0,215	Mid-Pleistocene Homo	All: Wu & Bruner 2016
Manot 1	1068	0,055	Homo sapiens	All: Weber et al., 2019
Malapa hominin 1 (MH1)	420	1,977	Australopithecines	Age & Taxon: Pickering et al., 2011, Endocranial Volume & Taxon: Berger et al., 2010
Minatogawa 1	1335	0,022	Homo sapiens	Endocranial Volume & Taxon: Kubo et al., 2012, Age & Taxon: Nakamura et al., 2019
Minatogawa 2	1170	0,022	Homo sapiens	Endocranial Volume & Taxon: Holloway, Broadfield, & Yuan, 2004, page 300, Age & Taxon: Nakamura et al., 2019
Minatogawa 4	1170	0,022	Homo sapiens	Endocranial Volume & Taxon: Kubo et al., 2012, Age & Taxon: Nakamura et al., 2019
Mladec 1	1606	0,036	Homo sapiens	Endocranial Volume & Taxon: Neubauer et al., 2018, Age & Taxon: Kuzmin & Keates, 2014
Mladec 2	1390	0,036	Homo sapiens	Age & Taxon: Kuzmin & Keates, 2014, Endocranial Volume & Taxon: Holloway, Broadfield, & Yuan, 2004, page 300
Mladec 5	1500	0,029	Homo sapiens	All: Shultz et al., 2012
MLD 1	510	2,715	Australopithecines	Endocranial Volume & Taxon: Shultz et al., 2012, Age & Taxon: Wood, 2011, page 481
MLD 37/38	440	2,580	Australopithecines	Endocranial Volume & Taxon: Neubauer et al., 2012, Age & Taxon: Wood, 2011, page 481
Monte Circeo (Guattari 1)	1350	0,053	Neanderthals	All: Recheis et al., 1999
Nanjing 1 (Nankin 1)	876	0,620	Erectines	Age & Taxon: Wood, 2011, page 770, Endocranial Volume & Taxon: Wu et al., 2011
Narmada 1 (Hathnora 1)	1249	0,236	Erectines	All: Shultz et al., 2012
Nazlet Khater 2	1420	0,038	Homo sapiens	Age: Grün, as cited in Crevecoeur et al., 2009, Endocranial Volume & Taxon: Shultz et al., 2012
Ndutu 1	1100	0,350	Mid-Pleistocene Homo	All: Shultz et al., 2012
Ngandong 1 (Solo I)	1121	0,113	Erectines	Age: Rizal et al., 2020, Taxon: Groves, 2013, Endocranial Volume: Shultz et al., 2012
Ngandong 13 (Solo X)	1109	0,113	Erectines	Age: Rizal et al., 2020, Taxon: Groves, 2013, Endocranial Volume: Shultz et al., 2012
Ngandong 14 (Solo XI)	1127	0,113	Erectines	Endocranial Volume: Neubauer et al., 2018, Age: Rizal et al., 2020, Taxon: Groves, 2013
Ngandong 5 (Solo IV)	1266	0,113	Erectines	Age: Rizal et al., 2020, Taxon: Groves, 2013, Endocranial Volume: Shultz et al., 2012
Ngandong 6 (Solo V)	1116	0,113	Erectines	Age: Rizal et al., 2020, Taxon: Groves, 2013, Endocranial Volume: Shultz et al., 2012
Ngandong 7 (Solo VI)	1028	0,113	Erectines	Age: Rizal et al., 2020, Endocranial Volume: Balzeau et al., 2003, Taxon: Groves, 2013
Ngandong 12 (Solo IX)	1135	0,113	Erectines	Age: Rizal et al., 2020, Taxon: Groves, 2013, Endocranial Volume: Shultz et al., 2012
Ngawi	952	0,113	Erectines	Endocranial Volume: Neubauer et al., 2018, Taxon: Groves, 2013, Age: Rizal et al., 2020
Oberkassel 1 (Oberkassel D998)	1492	0,013	Homo sapiens	All: Neubauer et al., 2018
Oberkassel 2 (Oberkassel D999)	1330	0,014	Homo sapiens	All: Neubauer et al., 2018
OH 12	732	1,100	Erectines	All: Shultz et al., 2012

OH 24	597	1,863	Habilines	Endocranial Volume & Taxon: Shultz et al., 2012, Age: Habermann et al., 2016 & Wood, 2011, page 566
OH 5	500	1,840	Paranthropines	Endocranial Volume & Taxon: Falk et al., 2000, Age: Habermann et al., 2016 & Wood, 2011, page 565
OH 7	777	1,840	Habilines	Endocranial Volume & Taxon: Spoor et al., 2015, Age: Habermann et al., 2016 & Wood, 2011, page 562
OH 9	1013	1,470	Erectines	Endocrancial Volume & Age: Neubauer et al., 2018, Taxon: Shultz et al., 2012
Omo 2 (Kibish)	1491	0,195	Homo sapiens	All: Neubauer et al., 2018
Omo L894-1	500	1,924	Habilines	Endocranial Volume & Taxon: Shultz et al., 2012, Age: Kidane et al., 2007 & Boaz & Howell, 1997
Pavlov 1	1522	0,030	Homo sapiens	Age & Taxon: Fewlass et al., 2019, Endocranial Volume & Taxon: Shultz et al., 2012
Petralona 1	1162	0,255	Mid-Pleistocene Homo	Endocranial Volume & Taxon: Neubauer et al., 2018, Age & Taxon: Shultz et al., 2012
Predmosti 10	1452	0,028	Homo sapiens	All: Shultz et al., 2012
Predmosti 3	1580	0,028	Homo sapiens	Age & Taxon: Shultz et al., 2012, Endocranial Volume & Taxon: Schwartz & Tattersall, 2002, page 300
Predmosti 4	1518	0,028	Homo sapiens	All: Shultz et al., 2012
Predmosti 9	1555	0,028	Homo sapiens	All: Shultz et al., 2012
Qafzeh 6	1524	0,115	Homo sapiens	All: Neubauer et al., 2018
Qafzeh 9	1497	0,115	Homo sapiens	All: Neubauer et al., 2018
Reilingen	1430	0,200	Mid-Pleistocene Homo	All: Holloway, Broadfield, & Yuan, 2004, page 298
Saccopastore 1	1174	0,245	Neanderthals	Age & Taxon: Marra et al., 2017, Endocranial Volume: Sergi, 1944
Saccopastore 2	1300	0,220	Neanderthals	Age & Taxon: Marra et al., 2017, Endocranial Volume & Taxon: Holloway, Broadfield, & Yuan, 2004, page 299
Saldanha 1	1217	0,800	Mid-Pleistocene Homo	All: Shultz et al., 2012
Sale 1	911	0,400	Erectines	All: Shultz et al., 2012
Sambungmacan 1 (Sm1)	1035	0,113	Erectines	Age & Taxon: Rizal et al., 2020, Endocranial Volume & Taxon: Holloway, Broadfield, & Yuan, 2004, page 298
Sambungmacan 3 (Poloyo) (Sm3)	902	0,113	Erectines	Endocranial Volume & Taxon: Neubauer et al., 2018, Age & Taxon: Rizal et al., 2020
Sambungmacan 4 (Sm4)	1006	0,113	Erectines	Age & Taxon: Rizal et al., 2020, Endocranial Volume & Taxon: Holloway, Broadfield, & Yuan, 2004, page 298
San Teodoro 1 (ST1)	1565	0,015	Homo sapiens	Age & Taxon: Mannino et al., 2011, Endocranial Volume & Taxon: Shultz et al., 2012
San Teodoro 2 (ST2)	1569	0,015	Homo sapiens	Age & Taxon: Mannino et al., 2011, Endocranial Volume & Taxon: Shultz et al., 2012
San Teodoro 3 (ST3)	1560	0,015	Homo sapiens	Age & Taxon: Mannino et al., 2011, Endocranial Volume & Taxon: Shultz et al., 2012
San Teodoro 5 (ST5)	1484	0,015	Homo sapiens	Age & Taxon: Garilli et al., 2020, Endocranial Volume & Taxon: Shultz et al., 2012
Sangiran 10	869	1,200	Erectines	Age & Taxon: Holloway, Broadfield, & Yuan, 2004, page 298, Endocranial Volume & Taxon: Shultz et al., 2012
Sangiran 12	951	0,900	Erectines	Age & Taxon: Holloway, Broadfield, & Yuan, 2004, page 298, Endocranial Volume & Taxon: Shultz et al., 2012
Sangiran 17	1020	1,000	Erectines	Age & Taxon: Holloway, Broadfield, & Yuan, 2004, page 298, Endocranial Volume & Taxon: Shultz et al., 2012
Sangiran 2	793	0,980	Erectines	Age & Taxon: Holloway, Broadfield, & Yuan, 2004, page 298, Endocranial Volume & Taxon: Neubauer et al., 2018
Sangiran 3	890	1,000	Erectines	Age & Taxon: Holloway, Broadfield, & Yuan, 2004, page 298, Endocranial Volume & Taxon: De Miguel & Henneberg, 2001
Sangiran 31	1000	0,900	Erectines	Age & Taxon: Holloway, Broadfield, & Yuan, 2004, page 298, Endocranial Volume & Taxon: Shultz et al., 2012
Sangiran 4	856	1,100	Erectines	Age & Taxon: Holloway, Broadfield, & Yuan, 2004, page 298, Endocranial Volume & Taxon: Shultz et al., 2012
Sangiran 9	850	1,100	Erectines	Age & Taxon: Holloway, Broadfield, & Yuan, 2004, page 298, Endocranial Volume & Taxon: Shultz et al., 2012
Shanidar 1	1600	0,050	Neanderthals	Age & Taxon: Pomeroy et al., 2020, Endocranial Volume & Taxon: Holloway, Broadfield, & Yuan, 2004, page 299
Shanidar 5	1550	0,050	Neanderthals	Age & Taxon: Pomeroy et al., 2020, Endocranial Volume & Taxon: Holloway, Broadfield, & Yuan, 2004, page 299
Singa 1	1550	0,133	Homo sapiens	All: Holloway, Broadfield, & Yuan, 2004, page 300
SK 1585	476	1,990	Paranthropines	Age: Gibbon et al., 2014 & Beaudet, 2019, Endocranial Volume & Taxon: Falk et al., 2000
SK 847	507	1,990	Habilines	Age: Gibbon et al., 2014 & Beaudet, 2019, Endocranial Volume & Taxon: Shultz et al., 2012
Skhul 4	1554	0,115	Homo sapiens	Age & Taxon: Shultz et al., 2012, Endocranial Volume: Holloway, Broadfield, & Yuan, 2004, page 299

Skhul 5	1363	0,098	Homo sapiens	Age & Taxon: Grün et al., 2005, Endocranial Volume & Taxon: Neubauer et al., 2018
Skhul 9	1587	0,131	Homo sapiens	Age & Taxon: Grün et al., 2005, Endocranial Volume & Taxon: Shultz et al., 2012
Spy I	1287	0,041	Neanderthals	Endocranial Volume & Taxon: Neubauer et al., 2018, Age & Taxon: Kuzmin & Keates, 2014
Spy II	1531	0,041	Neanderthals	Endocranial Volume & Taxon: Neubauer et al., 2018, Age & Taxon: Kuzmin & Keates, 2014
St. Germain-la-Rivière	1354	0,019	Homo sapiens	Age & Taxon: Kuzmin & Keates, 2014, Endocranial Volume & Taxon: Shultz et al., 2012
Steinheim 1	1140	0,225	Mid-Pleistocene Homo	Endocranial Volume & Taxon: Prossinger et al., 2003, Age & Taxon: Shultz et al., 2012
Sts 19/58	436	2,400	Australopithecines	Age & Taxon: Herries & Shaw, 2011, Endocranial Volume & Taxon: Shultz et al., 2012
Sts 5	475	2,100	Australopithecines	Endocranial Volume & Taxon: Neubauer et al., 2012, Age & Taxon: Herries & Shaw, 2011
Sts 60	390	2,400	Australopithecines	Endocranial Volume & Taxon: Neubauer et al., 2012, Age & Taxon: Herries & Shaw, 2011
Sts 71	412	2,400	Australopithecines	Endocranial Volume & Taxon: Neubauer et al., 2012, Age & Taxon: Herries & Shaw, 2011
Stw 505	569	2,400	Australopithecines	Endocranial Volume & Taxon: Neubauer et al., 2012, Age & Taxon: Herries & Shaw, 2011
Stw 53	570	1,650	Australopithecines	Age: Herries & Shaw, 2011, Taxon: Clarke, 2013, Endocranial Volume: Shultz et al., 2012
Stw 573	408	3,670	Australopithecines	All: Beaudet et al. 2019
Sungir 1	1511	0,031	Homo sapiens	Age & Taxon: Kuzmin et al., 2014, Endocranial Volume & Taxon: Trinkaus et al., 2014, page 92
Sungir 2	1418	0,031	Homo sapiens	Age & Taxon: Kuzmin et al., 2014, Endocranial Volume & Taxon: Holloway, Broadfield, & Yuan, 2004, page 300
Sungir 5	1432	0,031	Homo sapiens	Age & Taxon: Kuzmin et al., 2014, Endocranial Volume & Taxon: Holloway, Broadfield, & Yuan, 2004, page 300
Swanscombe	1305	0,400	Mid-Pleistocene Homo	All: Shultz et al., 2012
Tabun C1	1271	0,122	Neanderthals	All: Shultz et al., 2012
TM 266 (TM 266-01-060-1) ("Toumaï")	365	7,320	Early Hominins	Age & Taxon: Lebatard et al., 2010, Endocranial Volume & Taxon: Zollikofer et al., 2005
Trinil 2 (Java Man)	940	0,800	Erectines	Age: Ingicco et al., 2014, Endocranial Volume & Taxon: Shultz et al., 2012
Type 2	457	2,500	Australopithecines	All: Shultz et al., 2012
Vertesszöllös 2 ("Samu")	1335	0,315	Mid-Pleistocene Homo	Age & Taxon: Kele et al., 2015, Endocranial Volume & Taxon: Shultz et al., 2012
Veyrier 1	1430	0,015	Homo sapiens	All: Shultz et al., 2012
Xuchang 1	1800	0.115	Homo sapiens	All: Li et al. 2017;
Xujiayao 6	1700	0,180	Mid-Pleistocene Homo	All: Wu et al. 2022
Yinkou	1390	0,130	Homo sapiens	All: Holloway, Broadfield, & Yuan, 2004, page 300
Yunxian EV9002	1050	0,793	Erectines	Age & Taxon: Guo et al., 2013, Endocranial Volume & Taxon: Vialet et al., 2010
Zhoukodian I, D (Z 2)	1030	0,770	Erectines	Age & Taxon: Shen et al., 2009, Endocranial Volume & Taxon: Shultz et al., 2012
Zhoukoudian II, L (Z 11)	1015	0,770	Erectines	Age & Taxon: Shen et al., 2009, Endocranial Volume & Taxon: Shultz et al., 2012
Zhoukoudian I, L (Z 10)	1225	0,770	Erectines	Age & Taxon: Shen et al., 2009, Endocranial Volume & Taxon: Shultz et al., 2012
Zhoukoudian III, L (Z 12)	1030	0,770	Erectines	Age & Taxon: Shen et al., 2009, Endocranial Volume & Taxon: Shultz et al., 2012
Zhoukoudian III, H (Z 5)	1140	0,450	Erectines	Age & Taxon: Shen et al., 2001, Endocranial Volume & Taxon: Schwartz & Tattersall, 2003, page 547
Zhoukoudian VI (Z 6)	850	0,770	Erectines	Age & Taxon: Shen et al., 2009, Endocranial Volume & Taxon: Shultz et al., 2012
Zhoukoudian (Upper Cave) 1 (UC 101)	1500	0,036	Homo sapiens	Age & Taxon: Li et al., 2018, Endocranial Volume & Taxon: Holloway, Broadfield, & Yuan, 2004, page 300
Zhoukoudian (Upper Cave) 2 (UC 102)	1380	0,036	Homo sapiens	Age & Taxon: Li et al., 2018, Endocranial Volume & Taxon: Holloway, Broadfield, & Yuan, 2004, page 300
Zhoukoudian (Upper Cave) 3 (UC 103)	1290	0,036	Homo sapiens	Age & Taxon: Li et al., 2018, Endocranial Volume & Taxon: Holloway, Broadfield, & Yuan, 2004, page 300

Sources used for Cranial Capacity and Chronological Age Data

Alba, D. M. (2010). Cognitive inferences in fossil apes (Primates, Hominoidea): does encephalization reflect intelligence. Journal of Anthropological Sciences, 88(1), 11-48.

Arsuaga, J. L., Carretero, J. M., Lorenzo, C., Gracia, A., Martınez, I., De Castro, J. B., & Carbonell, E. (1997). Size variation in Middle Pleistocene humans. Science, 277(5329), 1086-1088. https://doi.org/10.1126/science.277.5329.1086

Baab, K. L., Rogers, M., Bruner, E., & Semaw, S. (2022). Reconstruction and analysis of the DAN5/P1 and BSN12/P1 Gona Early Pleistocene Homo fossils [Article]. Journal of Human Evolution, 162, Article 103102. https://doi.org/10.1016/j.jhevol.2021.103102

Bahain, J. J., Falgueres, C., Laurent, M., Dolo, J. M., Shao, Q., Auguste, P., & Tuffreau, A. (2015). ESR/U-series dating of faunal remains from the paleoanthropological site of Biache-Saint-Vaast (Pas-de-Calais, France). Quaternary Geochronology, 30, 541-546. https://doi.org/10.1016/j.quageo.2015.02.020

Balzeau, A., Grimaud-Hervé, D., Indriati, E., & Jacob, T. (2003). Computer tomography scanning of Homo erectus crania Ngandong 7 from Java: Internal structure, paleopathology and post-mortem history. Berkala Llmu Kedokteran, 35(3), 133-140

Barshay-Szmidt, C., Costamagno, S., Henry-Gambier, D., Laroulandie, V., Pétillon, J. M., Boudadi-Maligne, M., ... & Mallye, J. B. (2016). New extensive focused AMS 14C dating of the Middle and Upper Magdalenian of the western Aquitaine/Pyrenean region of France (ca. 19–14 ka cal BP): Proposing a new model for its chronological phases and for the timing of occupation. Quaternary International, 414, 62-91. https://doi.org/10.1016/j.quaint.2015.12.073

Beaudet, A. (2019). The inner ear of the Paranthropus specimen DNH 22 from Drimolen, South Africa. American journal of physical anthropology, 170(3), 439-446. https://doi.org/10.1002/ajpa.23901

Beaudet, A., Clarke, R. J., de Jager, E. J., Bruxelles, L., Carlson, K. J., Crompton, R., de Beer, F., Dhaene, J., Heaton, J. L., Jakata, K., Jashashvili, T., Kuman, K., McClymont, J., Pickering, T. R., & Stratford, D. (2019). The endocast of StW 573 (“Little Foot”) and hominin brain evolution [Article]. Journal of Human Evolution, 126, 112-123. https://doi.org/10.1016/j.jhevol.2018.11.009

Benazzi, S., Gruppioni, G., Strait, D. S., & Hublin, J. J. (2014). Virtual reconstruction of KNM-ER 1813 Homo habilis cranium. American journal of physical anthropology, 153(1), 154-160. https://doi.org/10.1002/ajpa.22376

Berger, L. R., Hawks, J., de Ruiter, D. J., Churchill, S. E., Schmid, P., Delezene, L. K., Kivell, T. L., Garvin, H. M., Williams, S. A., DeSilva, J. M., Skinner, M. M., Musiba, C. M., Cameron, N., Holliday, T. W., Harcourt-Smith, W., Ackermann, R. R., Bastir, M., Bogin, B., Bolter, D., … Zipfel, B. (2015). Homo Naledi, a new species of the genus homo from the Dinaledi Chamber, South Africa. ELife, 4, Article e09560. https://doi.org/10.7554/elife.09560

Berger, L. R., De Ruiter, D. J., Churchill, S. E., Schmid, P., Carlson, K. J., Dirks, P. H., & Kibii, J. M. (2010). Australopithecus sediba: a new species of Homo-like australopith from South Africa. Science, 328(5975), 195-204. https://doi.org/10.1126/science.1184944

Bermúdez de Castro, J. M., Arsuaga, J. L., Carbonell, E., Rosas, A., Martınez, I., & Mosquera, M. (1997). A hominid from the Lower Pleistocene of Atapuerca, Spain: possible ancestor to Neandertals and modern humans. Science, 276(5317), 1392-1395. https://doi.org/10.1126/science.276.5317.1392

Boaz, N. T., & Clark Howell, F. (1977). A gracile hominid cranium from Upper Member G of the Shungura Formation, Ethiopia. American Journal of Physical Anthropology, 46(1), 93-108. https://doi.org/10.1002/ajpa.1330460113

Bräuer, G., Groden, C., Gröning, F., Kroll, A., Kupczik, K., Mbua, E., ... & Schiemann, T. (2004). Virtual study of the endocranial morphology of the matrix-filled cranium from Eliye Springs, Kenya. The Anatomical Record Part A: Discoveries in Molecular, Cellular, and Evolutionary Biology: An Official Publication of the American Association of Anatomists, 276(2), 113-133. https://doi.org/10.1002/ar.a.90122

Bräuer, G., Yokoyama, Y., Falguères, C., & Mbua, E. (1997). Modern human origins backdated. Nature, 386(6623), 337-338.

Brown, B., Walker, A., Ward, C. V., & Leakey, R. E. (1993). New Australopithecus boisei calvaria from East Lake Turkana, Kenya. American Journal of Physical Anthropology, 91(2), 137-159. https://doi.org/10.1002/ajpa.1330910202

Bruner, E., Bondioli, L., Coppa, A., Frayer, D. W., Holloway, R. L., Libsekal, Y., ... & Macchiarelli, R. (2016). The endocast of the one-million-year-old human cranium from Buia (UA 31), Danakil Eritrea. American journal of physical anthropology, 160(3), 458-468. https://doi.org/10.1002/ajpa.22983

Bruner, E., Grimaud-Hervé, D., Wu, X., de la Cuétara, J. M., & Holloway, R. (2015). A paleoneurological survey of Homo erectus endocranial metrics. Quaternary International, 368, 80-87. https://doi.org/10.1016/j.quaint.2014.10.007

Clarke, R. (2013) Australopithecus from Sterkfontein Caves, South Africa. In K. Reed, J. Fleagle, R. Leakey (Eds), The Paleobiology of Australopithecus. Vertebrate Paleobiology and Paleoanthropology (pp. 21-40). Springer. https://doi.org/10.1007/978-94-007-5919-0_7

Cofran, Z., Boone, M., & Petticord, M. (2021). Virtually estimated endocranial volumes of the krapina neandertals. American Journal of Physical Anthropology, 174(1), 117–128. https://doi.org/10.1002/ajpa.24165

Crevecoeur, I., Rougier, H., Grine, F., & Froment, A. (2009). Modern human cranial diversity in the Late Pleistocene of Africa and Eurasia: evidence from Nazlet Khater, Peştera cu Oase, and Hofmeyr. American Journal of Physical Anthropology, 140(2), 347-358. https://doi.org/10.1002/ajpa.21080

De Miguel, C., & Henneberg, M. (2001). Variation in hominid brain size: How much is due to method? HOMO, 52(1), 3–58. https://doi.org/10.1078/0018-442x-00019

Demuro, M., Arnold, L. J., Aranburu, A., Sala, N., & Arsuaga, J. L. (2019). New bracketing luminescence ages constrain the Sima de los Huesos hominin fossils (Atapuerca, Spain) to MIS 12. Journal of human evolution, 131, 76-95. https://doi.org/10.1016/j.jhevol.2018.12.003

Dinnis, R., Bessudnov, A., Reynolds, N., Devièse, T., Pate, A., Sablin, M., ... & Higham, T. (2019). New data for the early Upper Paleolithic of Kostenki (Russia). Journal of Human Evolution, 127, 21-40. https://doi.org/10.1016/j.jhevol.2018.11.012

Dirks, P. H., Roberts, E. M., Hilbert-Wolf, H., Kramers, J. D., Hawks, J., Dosseto, A., ... & Hellstrom, J. (2017). The age of Homo naledi and associated sediments in the Rising Star Cave, South Africa. Elife, 6, e24231. https://doi.org/10.7554/eLife.24231

Falguères, C., Shao, Q., Han, F., Bahain, J. J., Richard, M., Perrenoud, C., & Moigne, A. M. (2015). New ESR and U-series dating at Caune de l'Arago, France: A key-site for European Middle Pleistocene. Quaternary Geochronology, 30, 547-553. https://doi.org/10.1016/j.quageo.2015.02.006

Falk, D., Redmond Jr, J. C., Guyer, J., Conroy, C., Recheis, W., Weber, G. W., & Seidler, H. (2000). Early hominid brain evolution: a new look at old endocasts. Journal of Human Evolution, 38(5), 695-717. https://doi.org/10.1006/jhev.1999.0378

Feibel, C. S., Lepre, C. J., & Quinn, R. L. (2009). Stratigraphy, correlation, and age estimates for fossils from Area 123, Koobi Fora. Journal of Human Evolution, 57(2), 112-122. https://doi.org/10.1016/j.jhevol.2009.05.007

Ferring, R., Oms, O., Agustí, J., Berna, F., Nioradze, M., Shelia, T., ... & Lordkipanidze, D. (2011). Earliest human occupations at Dmanisi (Georgian Caucasus) dated to 1.85–1.78 Ma. Proceedings of the National Academy of Sciences, 108(26), 10432-10436. https://doi.org/10.1073/pnas.1106638108

Fewlass, H., Talamo, S., Kromer, B., Bard, E., Tuna, T., Fagault, Y., ... & Sázelová, S. (2019). Direct radiocarbon dates of mid Upper Palaeolithic human remains from Dolní Věstonice II and Pavlov I, Czech Republic. Journal of Archaeological Science: Reports, 27, 102000. https://doi.org/10.1016/j.jasrep.2019.102000

Garilli, V., Vita, G., Mulone, A., Bonfiglio, L., & Sineo, L. (2020). From sepulchre to butchery-cooking: facies analysis, taphonomy and stratigraphy of the Upper Palaeolithic post burial layer from the San Teodoro Cave (NE Sicily) reveal change in the use of the site. Journal of Archaeological Science: Reports, 30, 102191. https://doi.org/10.1016/j.jasrep.2020.102191

Ghinassi, M., Oms, O., Papini, M., Scarciglia, F., Carnevale, G., Sani, F., ... & Bondioli, L. (2015). An integrated study of the Homo-bearing Aalat stratigraphic section (Eritrea): An expanded continental record at the Early–Middle Pleistocene transition. Journal of African Earth Sciences, 112, 163-185. https://doi.org/10.1016/j.jafrearsci.2015.09.012

Gibbon, R. J., Pickering, T. R., Sutton, M. B., Heaton, J. L., Kuman, K., Clarke, R. J., ... & Granger, D. E. (2014). Cosmogenic nuclide burial dating of hominin-bearing Pleistocene cave deposits at Swartkrans, South Africa. Quaternary Geochronology, 24, 10-15. https://doi.org/10.1016/j.quageo.2014.07.004

Groves, C. (2013). Hominin migrations before Homo sapiens: Out of Africa–how many times?. In Ness, I., & Bellwood, P. S. (Eds.), The encyclopedia of global human migration (pp. 18-22). Wiley-Blackwell. https://doi.org/10.1002/9781444351071.wbeghm803

Grün, R., Pike, A., McDermott, F., Eggins, S., Mortimer, G., Aubert, M., ... & Brink, J. (2020). Dating the skull from Broken Hill, Zambia, and its position in human evolution. Nature, 580(7803), 372-375. https://doi.org/10.1038/s41586-020-2165-4

Guérin, G., Frouin, M., Talamo, S., Aldeias, V., Bruxelles, L., Chiotti, L., ... & Lahaye, C. (2015). A multi-method luminescence dating of the Palaeolithic sequence of La Ferrassie based on new excavations adjacent to the La Ferrassie 1 and 2 skeletons. Journal of Archaeological Science, 58, 147-166. https://doi.org/10.1016/j.jas.2015.01.019

Gunz, P., Neubauer, S., Falk, D., Tafforeau, P., Le Cabec, A., Smith, T. M., ... & Alemseged, Z. (2020). Australopithecus afarensis endocasts suggest ape-like brain organization and prolonged brain growth. Science advances, 6(14), eaaz4729. https://doi.org/10.1126/sciadv.aaz4729

Guipert, G., de Lumley, M. A., Tuffreau, A., & Mafart, B. (2011). A late Middle Pleistocene hominid: Biache-Saint-Vaast 2, north France. Comptes Rendus Palevol, 10(1), 21-33. https://doi.org/10.1016/j.crpv.2010.10.006

Guo, Y., Huang, C. C., Pang, J., Zha, X., Zhou, Y., Zhang, Y., & Zhou, L. (2013). Sedimentological study of the stratigraphy at the site of Homo erectus yunxianensis in the upper Hanjiang River valley, China. Quaternary International, 300, 75-82. https://doi.org/10.1016/j.quaint.2012.12.036

Grimaud-Hervé, D., Albessard-Ball, L., Pokhojaev, A., Balzeau, A., Sarig, R., Latimer, B., McDermott, Y., May, H., & Hershkovitz, I. (in press). The endocast of the Late Middle Paleolithic Manot 1 specimen, Western Galilee, Israel. Journal of Human Evolution. https://doi.org/10.1016/j.jhevol.2019.102734

Grün, R., Stringer, C., McDermott, F., Nathan, R., Porat, N., Robertson, S., ... & McCulloch, M. (2005). U-series and ESR analyses of bones and teeth relating to the human burials from Skhul. Journal of human evolution, 49(3), 316-334. https://doi.org/10.1016/j.jhevol.2005.04.006

Habermann, J. M., Stanistreet, I. G., Stollhofen, H., Albert, R. M., Bamford, M. K., Pante, M. C., ... & Masao, F. T. (2016). In situ∼ 2.0 Ma trees discovered as fossil rooted stumps, lowermost Bed I, Olduvai Gorge, Tanzania. Journal of human evolution, 90, 74-87. https://doi.org/10.1016/j.jhevol.2015.09.011

Herries, A. I., & Shaw, J. (2011). Palaeomagnetic analysis of the Sterkfontein palaeocave deposits: Implications for the age of the hominin fossils and stone tool industries. Journal of Human Evolution, 60(5), 523-539. https://doi.org/10.1016/j.jhevol.2010.09.001

Hoffmann, A., Hublin, J. J., Hüls, M., & Terberger, T. (2011). The Homo aurignaciensis hauseri from Combe-Capelle–a Mesolithic burial. Journal of Human Evolution, 61(2), 211-214. https://doi.org/10.1016/j.jhevol.2011.03.001

Holloway, R. L., Broadfield, D. C., & Yuan, M. S. (2004). The Human Fossil Record. Volume 3: Brain Endocasts--The Paleoneurological Evidence. John Wiley & Sons.

Ingicco, T., de Vos, J., & Huffman, O. F. (2014). The oldest gibbon fossil (Hylobatidae) from insular Southeast Asia: Evidence from Trinil,(East Java, Indonesia), Lower/Middle Pleistocene. PloS one, 9(6), e99531. https://doi.org/10.1371/journal.pone.0099531

Ji, Q., Wu, W., Ji, Y., Li, Q., & Ni, X. (2021). Late Middle Pleistocene Harbin cranium represents a new Homo species [Note]. Innovation, 2(3), Article 100132. https://doi.org/10.1016/j.xinn.2021.100132

Kele, S., Markó, A., Kisné Cseh, J., CHuan-CHou, S., Chung-CHe, W., & Bernasconi, A. M. (2015). Samuel's paleo-jacuzzi: Dating and clumped isotope-based temperature of the Vértesszőlős Early Man site (Hungary). In Abstracts of 31st IAS Meeting of Sedimentology held in Krakow on 22nd–25th of June 2015 (p. 268). Polish Geological Society. Available online at http://www.ing.uj.edu.pl/ims2015/

Keen, J. A. (1942). Report on a Skeleton from the Fish Hoek Cave. South African Journal of Science, 38, 301-309.

Kidane, T., Otofuji, Y. I., Brown, F. H., Takemoto, K., & Eshete, G. (2007). Two normal paleomagnetic polarity intervals in the lower Matuyama Chron recorded in the Shungura Formation (Omo Valley, Southwest Ethiopia). Earth and Planetary Science Letters, 262(1-2), 240-256. https://doi.org/10.1016/j.epsl.2007.07.047

Kimbel, W. H. (1984). Variation in the pattern of cranial venous sinuses and hominid phylogeny. American Journal of Physical Anthropology, 63(3), 243-263. https://doi.org/10.1002/ajpa.1330630302

Kranioti, E. F., Holloway, R., Senck, S., Ciprut, T., Grigorescu, D., & Harvati, K. (2011). Virtual assessment of the endocranial morphology of the early modern European fossil calvaria from Cioclovina, Romania [Article]. Anatomical Record, 294(7), 1083-1092. https://doi.org/10.1002/ar.21420

Kubo, D., Kono, R. T., & Kaifu, Y. (2013). Brain size of Homo floresiensis and its evolutionary implications. Proceedings of the Royal Society B: Biological Sciences, 280(1760), 20130338. https://doi.org/10.1098/rspb.2013.0338

Kubo, D., Kono, R. T., & Suwa, G. (2012). Endocranial proportions and postorbital morphology of the Minatogawa I and IV Late Pleistocene Homo sapiens crania from Okinawa Island, Japan. Anthropological Science, 1203090132-1203090132. https://doi.org/10.1537/ase.110804

Kuhn, B. F., Herries, A. I. R., Price, G., Baker, S. E., Hopley, P. J., Menter, C., & Caruana, M. V. (2016). Renewed investigations at Taung; 90 years after the discovery of Australopithecus africanus. Palaeontologia africana, 51, 10-26.

Kuzmin, Y. V., & Keates, S. G. (2014). Direct radiocarbon dating of Late Pleistocene hominids in Eurasia: current status, problems, and perspectives. Radiocarbon, 56(2), 753-766. https://doi.org/10.2458/56.16936

Kuzmin, Y. V., van der Plicht, J., & Sulerzhitsky, L. D. (2014). Puzzling radiocarbon dates for the Upper Paleolithic site of Sungir (central Russian Plain). Radiocarbon, 56(2), 451-459. https://doi.org/10.2458/56.17038

Lebatard, A. E., Bourlès, D. L., Braucher, R., Arnold, M., Duringer, P., Jolivet, M., ... & Schuster, M. (2010). Application of the authigenic 10Be/9Be dating method to continental sediments: reconstruction of the Mio-Pleistocene sedimentary sequence in the early hominid fossiliferous areas of the northern Chad Basin. Earth and Planetary Science Letters, 297(1-2), 57-70. https://doi.org/10.1016/j.epsl.2010.06.003

Lepre, C. J., & Kent, D. V. (2015). Chronostratigraphy of KNM-ER 3733 and other Area 104 hominins from Koobi Fora. Journal of human evolution, 86, 99-111. https://doi.org/10.1016/j.jhevol.2015.06.010

Li, F., Bae, C. J., Ramsey, C. B., Chen, F., & Gao, X. (2018). Re-dating Zhoukoudian Upper Cave, northern China and its regional significance. Journal of human evolution, 121, 170-177. https://doi.org/10.1016/j.jhevol.2018.02.011

Li, Z. Y., Wu, X. J., Zhou, L. P., Liu, W., Gao, X., Nian, X. M., & Trinkaus, E. (2017). Late Pleistocene archaic human crania from Xuchang, China [Article]. Science, 355(6328), 969-972. https://doi.org/10.1126/science.aal2482

Lordkipanidze, D., de León, M. S. P., Margvelashvili, A., Rak, Y., Rightmire, G. P., Vekua, A., & Zollikofer, C. P. (2013). A complete skull from Dmanisi, Georgia, and the evolutionary biology of early Homo. Science, 342(6156), 326-331. https://doi.org/10.1126/science.1238484

Mannino, M. A., Di Salvo, R., Schimmenti, V., Di Patti, C., Incarbona, A., Sineo, L., & Richards, M. P. (2011). Upper Palaeolithic hunter-gatherer subsistence in Mediterranean coastal environments: an isotopic study of the diets of the earliest directly-dated humans from Sicily. Journal of Archaeological Science, 38(11), 3094-3100. https://doi.org/10.1016/j.jas.2011.07.009

Marra, F., Ceruleo, P., Pandolfi, L., Petronio, C., Rolfo, M. F., & Salari, L. (2017). The aggradational successions of the Aniene River Valley in Rome: age constraints to early neanderthal presence in Europe. PloS one, 12(1), e0170434. https://doi.org/10.1371/journal.pone.0170434

McDougall, I., Brown, F. H., Vasconcelos, P. M., Cohen, B. E., Thiede, D. S., & Buchanan, M. J. (2012). New single crystal 40Ar/39Ar ages improve time scale for deposition of the Omo Group, Omo–Turkana Basin, East Africa. Journal of the Geological Society, 169(2), 213-226. https://doi.org/10.1144/0016-76492010-188

Nakamura, T., Terada, T., Ueki, C., & Minami, M. (2019). Radiocarbon dating of textile Components from Historical silk costumes and other cloth products in the Ryukyu Islands, Japan. Radiocarbon, 61(6), 1663-1674. https://doi.org/10.1017/RDC.2019.105

Neubauer, S., Gunz, P., Weber, G. W., & Hublin, J. J. (2012). Endocranial volume of Australopithecus africanus: new CT-based estimates and the effects of missing data and small sample size. Journal of Human Evolution, 62(4), 498-510. https://doi.org/10.1016/j.jhevol.2012.01.005

Neubauer, S., Hublin, J. J., & Gunz, P. (2018). The evolution of modern human brain shape. Science advances, 4(1), eaao5961. https://doi.org/10.1126/sciadv.aao5961

Neubauer, S. (2022). The Endocast of the Late Pleistocene Human Skull from Hofmeyr. In Vertebrate Paleobiology and Paleoanthropology (pp. 153-163).

Pickering, R., Dirks, P. H., Jinnah, Z., De Ruiter, D. J., Churchill, S. E., Herries, A. I., ... & Berger, L. R. (2011). Australopithecus sediba at 1.977 Ma and implications for the origins of the genus Homo. science, 333(6048), 1421-1423. https://doi.org/10.1126/science.1203697

Pickering, T. R., Domínguez-Rodrigo, M., Egeland, C. P., & Brain, C. K. (2004). Beyond leopards: tooth marks and the contribution of multiple carnivore taxa to the accumulation of the Swartkrans Member 3 fossil assemblage. Journal of Human Evolution, 46(5), 595-604. https://doi.org/10.1016/j.jhevol.2004.03.002

Pomeroy, E., Bennett, P., Hunt, C. O., Reynolds, T., Farr, L., Frouin, M., ... & Barker, G. (2020). New Neanderthal remains associated with the 'flower burial'at Shanidar Cave. Antiquity, 94(373), 11-26. https://doi.org/10.15184/aqy.2019.207

Poza-Rey, E. M., Gómez-Robles, A., & Arsuaga, J. L. (2019). Brain size and organization in the Middle Pleistocene hominins from Sima de los Huesos. Inferences from endocranial variation. Journal of human evolution, 129, 67-90. https://doi.org/10.1016/j.jhevol.2019.01.006

Prossinger, H., Seidler, H., Wicke, L., Weaver, D., Recheis, W., Stringer, C., & Müller, G. B. (2003). Electronic removal of encrustations inside the Steinheim cranium reveals paranasal sinus features and deformations, and provides a revised endocranial volume estimate. The Anatomical Record Part B: The New Anatomist: An Official Publication of the American Association of Anatomists, 273(1), 132-142. https://doi.org/10.1002/ar.b.10022

Recheis, W., Macchiarelli, R., Seidler, H., Weaver, D. S., Schäfer, K., Bondioli, L., Weber, G. W., & zur Nedden, D. (1999). Re-evaluation of the endocranial volume of the Guattari 1 Neandertal specimen (Monte Circeo). Collegium antropologicum, 23(2), 397–405.

Renne, P. R., WoldeGabriel, G., Hart, W. K., Heiken, G., & White, T. D. (1999). Chronostratigraphy of the Miocene–Pliocene Sagantole Formation, Middle Awash Valley, Afar rift, Ethiopia. Geological Society of America Bulletin, 111(6), 869-885. https://doi.org/10.1130/0016-7606(1999)111<0869:COTMPS>2.3.CO;2

Rightmire, G. P., de León, M. S. P., Lordkipanidze, D., Margvelashvili, A., & Zollikofer, C. P. (2017). Skull 5 from Dmanisi: Descriptive anatomy, comparative studies, and evolutionary significance. Journal of Human Evolution, 104, 50-79. https://doi.org/10.1016/j.jhevol.2017.01.005

Rizal, Y., Westaway, K. E., Zaim, Y., van den Bergh, G. D., Bettis, E. A., Morwood, M. J., ... & Westaway, M. C. (2020). Last appearance of Homo erectus at Ngandong, Java, 117,000–108,000 years ago. Nature, 577(7790), 381-385. https://doi.org/10.1038/s41586-019-1863-2

Schwartz, J. H. (2002). The Human Fossil Record. Volume 1: Terminology and Craniodental Morphology. John Wiley & Sons.

Schwartz, J. H., & Tattersall, I. (2003). The human fossil record. Volume 2: craniodental morphology of genus Homo (Africa and Asia). John Wiley & Sons.

Schwartz, J. H., & Tattersall, I. (2005). The Human Fossil Record. Volume 4: Craniodental Morphology of Early Hominids (Genera, Australopithecus, Paranthropus, Orrorin), and Overview. John Wiley & Sons.

Sergi, S. (1944). Craniometria e craniografia del primo paleantropo di Saccopastore. Ricerche di Morfologia, 20-21, 733-791.

Shao, Q., Ge, J., Ji, Q., Li, J., Wu, W., Ji, Y., Zhan, T., Zhang, C., Li, Q., Grün, R., Stringer, C., & Ni, X. (2021). Geochemical provenancing and direct dating of the Harbin archaic human cranium [Article]. Innovation, 2(3), Article 100131. https://doi.org/10.1016/j.xinn.2021.100131

Shen, G., Gao, X., Gao, B., & Granger, D. E. (2009). Age of Zhoukoudian Homo erectus determined with 26 Al/10 Be burial dating. Nature, 458(7235), 198-200. https://doi.org/10.1038/nature07741

Shen, G., Ku, T. L., Cheng, H., Edwards, R. L., Yuan, Z., & Wang, Q. (2001). High-precision U-series dating of Locality 1 at Zhoukoudian, China. Journal of human evolution, 41(6), 679-688. https://doi.org/10.1006/jhev.2001.0516

Shultz, S., Nelson, E., & Dunbar, R. I. (2012). Hominin cognitive evolution: identifying patterns and processes in the fossil and archaeological record. Philosophical Transactions of the Royal Society B: Biological Sciences, 367(1599) (Suppl.). https://doi.org/10.1098/rstb.2012.0115

Spoor, F., Gunz, P., Neubauer, S., Stelzer, S., Scott, N., Kwekason, A., & Dean, M. C. (2015). Reconstructed Homo habilis type OH 7 suggests deep-rooted species diversity in early Homo. Nature, 519(7541), 83-86. https://doi.org/10.1038/nature14224

Spoor, F., Leakey, M. G., Gathogo, P. N., Brown, F. H., Antón, S. C., McDougall, I., ... & Leakey, L. N. (2007). Implications of new early Homo fossils from Ileret, east of Lake Turkana, Kenya. Nature, 448(7154), 688-691. https://doi.org/10.1038/nature05986

Stynder, D. D., Brock, F., Sealy, J. C., Wurz, S., Morris, A. G., & Volman, T. P. (2009). A mid-Holocene AMS 14C date for the presumed upper Pleistocene human skeleton from Peers Cave, South Africa [Article]. Journal of Human Evolution, 56(4), 431-434. https://doi.org/10.1016/j.jhevol.2008.11.004

Sun, X., Yi, S., Lu, H., & Zhang, W. (2017). TT-OSL and post-IR IRSL dating of the Dali Man site in central China. Quaternary International, 434, 99-106. https://doi.org/10.1016/j.quaint.2015.05.027

Sutikna, T., Tocheri, M. W., Morwood, M. J., Saptomo, E. W., Awe, R. D., Wasisto, S., ... & Storey, M. (2016). Revised stratigraphy and chronology for Homo floresiensis at Liang Bua in Indonesia. Nature, 532(7599), 366-369. https://doi.org/10.1038/nature17179

Suwa, G., Asfaw, B., Kono, R. T., Kubo, D., Lovejoy, C. O., & White, T. D. (2009). The Ardipithecus ramidus skull and its implications for hominid origins. Science, 326(5949), 68-68e7. https://doi.org/10.1126/science.1175825

Trinkaus, E., Buzhilova, A. P., Mednikova, M. B., & Dobrovolskaya, M. V. (2014). The people of Sunghir: Burials, bodies, and behavior in the earlier Upper Paleolithic. Oxford University Press.

Vialet, A., Guipert, G., Jianing, H., Xiaobo, F., Zune, L., Youping, W., ... & de Lumley, H. (2010). Homo erectus from the Yunxian and Nankin Chinese sites: Anthropological insights using 3D virtual imaging techniques. Comptes Rendus Palevol, 9(6-7), 331-339. https://doi.org/10.1016/j.crpv.2010.07.017

Weber, G. W., Hershkovitz, I., Gunz, P., Neubauer, S., Ayalon, A., Latimer, B., ... & May, H. (2019). Before the massive modern human dispersal into Eurasia: A 55,000-year-old partial cranium from Manot Cave, Israel. Quaternary International. https://doi.org/10.1016/j.quaint.2019.10.009

Wood, B. (Ed.). (2011). Wiley-Blackwell encyclopedia of human evolution. John Wiley & Sons.

Wu, X., Holloway, R. L., Schepartz, L. A., & Xing, S. (2011). A new brain endocast of Homo erectus from Hulu Cave, Nanjing, China. American journal of physical anthropology, 145(3), 452-460. https://doi.org/10.1002/ajpa.21527

Wu, X., Liu, W., Dong, W., Que, J., & Wang, Y. (2008). The brain morphology of Homo Liujiang cranium fossil by three-dimensional computed tomography. Chinese Science Bulletin, 53(16), 2513-2519. https://doi.org/10.1007/s11434-008-0263-z

Wu, X. J., & Bruner, E. (2016). The endocranial anatomy of maba 1 [Article]. American Journal of Physical Anthropology, 160(4), 633-643. https://doi.org/10.1002/ajpa.22974

Wu, X. J., Pei, S. W., Cai, Y. J., Tong, H. W., Li, Q., Dong, Z., Sheng, J. C., Jin, Z. T., Ma, D. D., Xing, S., Li, X. L., Cheng, X., Cheng, H., de la Torre, I., Lawrence Edwards, R., Gong, X. C., An, Z. S., Trinkaus, E., & Liu, W. (2019). Archaic human remains from Hualongdong, China, and Middle Pleistocene human continuity and variation [Article]. Proceedings of the National Academy of Sciences of the United States of America, 116(20), 9820-9824. https://doi.org/10.1073/pnas.1902396116

Wu, X. J., Bae, C. J., Friess, M., Xing, S., Athreya, S., & Liu, W. (2022). Evolution of cranial capacity revisited: A view from the late Middle Pleistocene cranium from Xujiayao, China [Article]. Journal of Human Evolution, 163, Article 103119. https://doi.org/10.1016/j.jhevol.2021.103119

Zhu, Z. Y., Dennell, R., Huang, W. W., Wu, Y., Rao, Z. G., Qiu, S. F., ... & Zhou, H. Y. (2015). New dating of the Homo erectus cranium from Lantian (Gongwangling), China. Journal of Human Evolution, 78, 144-157. https://doi.org/10.1016/j.jhevol.2014.10.001

Zollikofer, C. P., de León, M. S. P., Lieberman, D. E., Guy, F., Pilbeam, D., Likius, A., ... & Brunet, M. (2005). Virtual cranial reconstruction of Sahelanthropus tchadensis. Nature, 434(7034), 755-759. https://doi.org/10.1038/nature03397

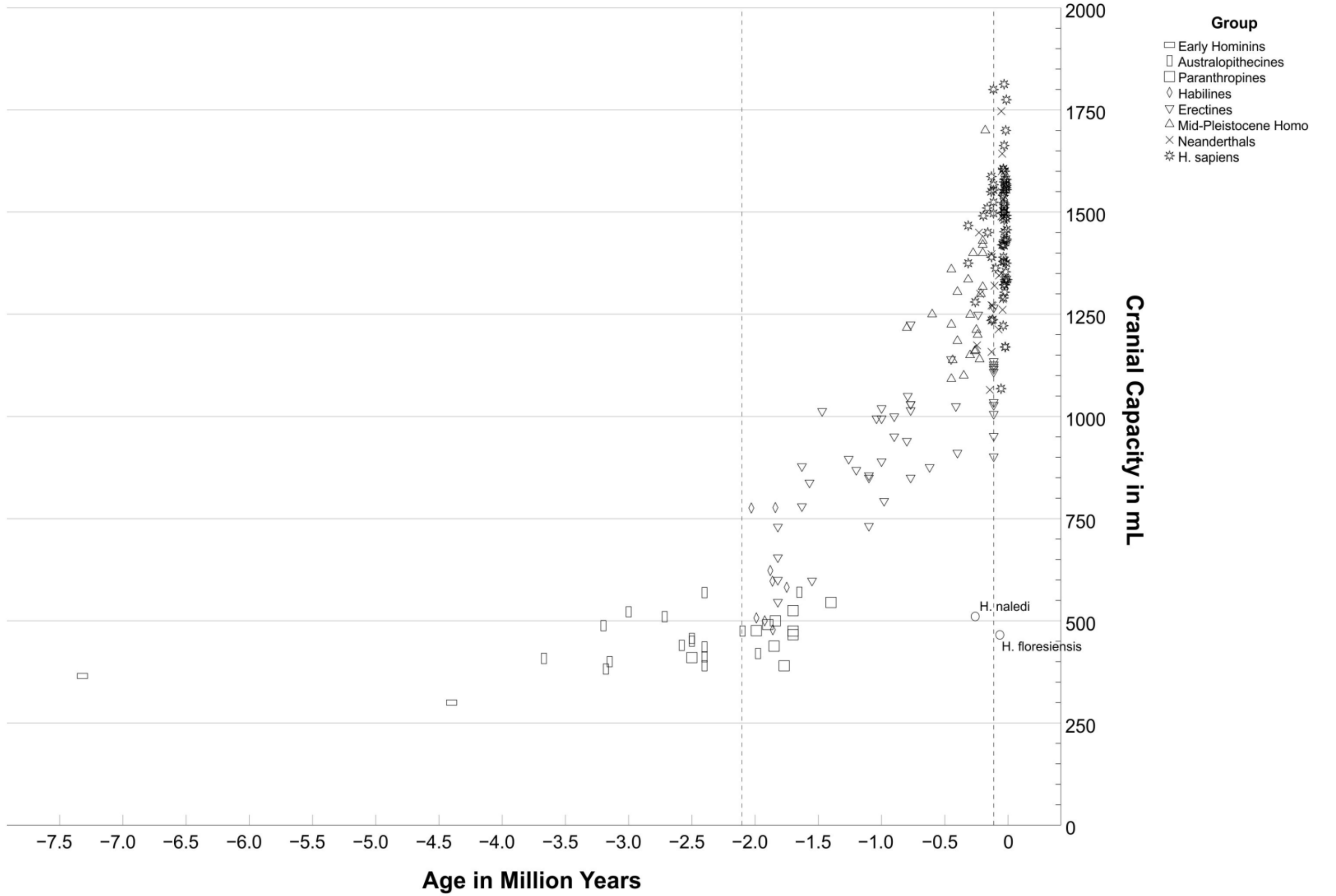

Figure S1: Cranial capacity in mL of 193 hominin fossils from the Late Miocene to the Late Pleistocene. Results are the same as in Figure 1 of the main text, but specimens were grouped according to their taxonomic affiliation here into eight larger clusters (see Section 2 for more explanations). *H. naledi* and *H. floresiensis* are also plotted to demonstrate their particular state (see Section 2).

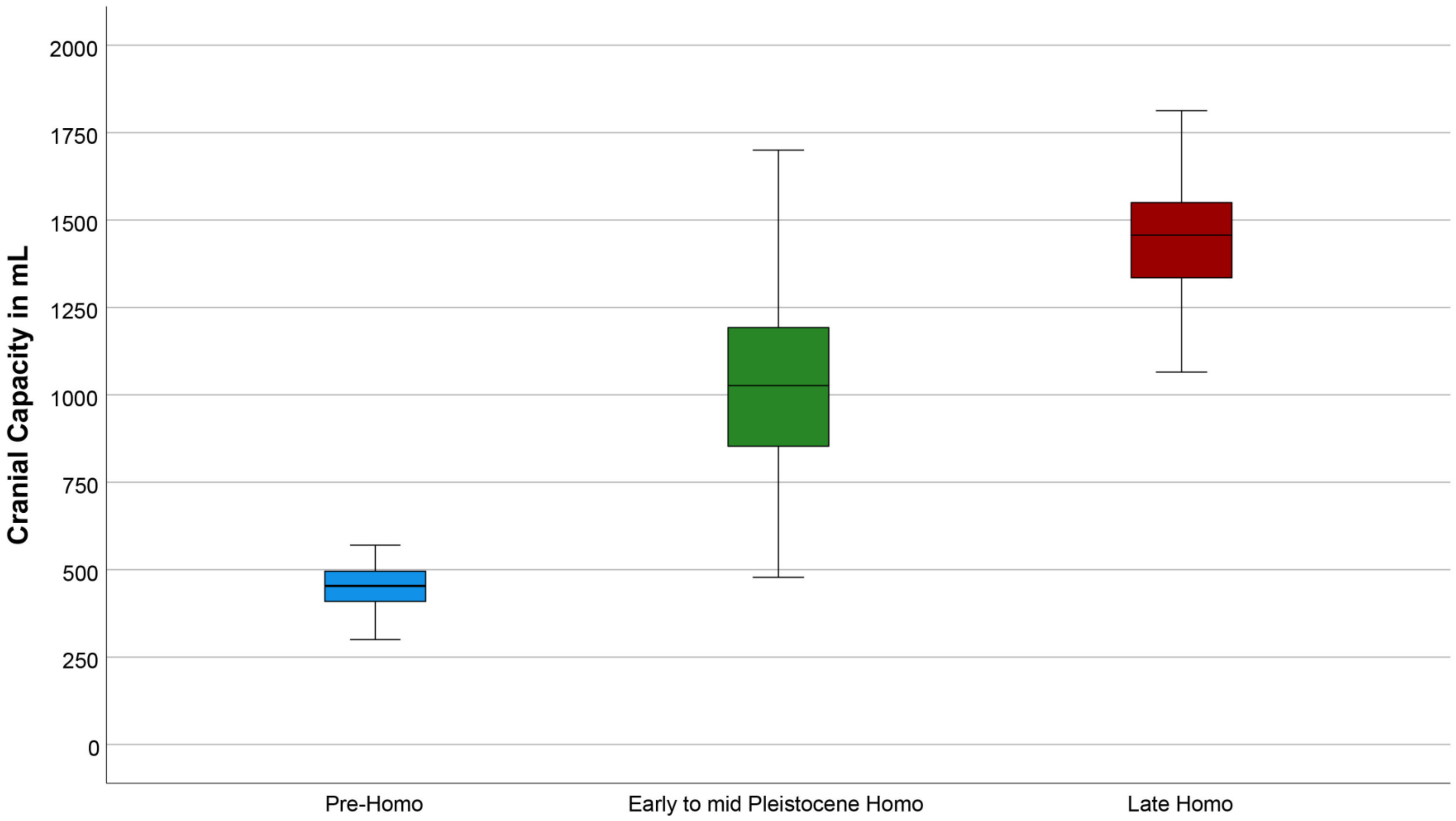

Figure S2: Boxplot of cranial capacities for the three hominin super-groups. Note there is an increase in the median of cranial capacity from one group to the next of roughly ~ 500mL
